# Membrane Heteroreceptor Complexes as Second-Order Protein Modulators: A Novel Integrative Mechanism through Allosteric Receptor–Receptor Interactions

**DOI:** 10.3390/membranes14050096

**Published:** 2024-04-25

**Authors:** Marina Mirchandani-Duque, Malak Choucri, Juan C. Hernández-Mondragón, Minerva Crespo-Ramírez, Catalina Pérez-Olives, Luca Ferraro, Rafael Franco, Miguel Pérez de la Mora, Kjell Fuxe, Dasiel O. Borroto-Escuela

**Affiliations:** 1 Receptomics and Brain Disorders Lab, Department of Human Physiology Physical Education and Sport, Faculty of Medicine, University of Malaga, 29010 Málaga, Spain; marina.md97@uma.es; 2 Department of Neuroscience, Karolinska Institutet, Biomedicum (B0852), Solnavägen 9, 17165 Solna, Sweden; malak.choucri@biology.ox.ac.uk; 3 Instituto de Fisiología Celular, Universidad Nacional Autónoma de México, Mexico City 04510, Mexico; jcmondragon@ifc.unam.mx (J.C.H.-M.); mcrespo@ifc.unam.mx (M.C.-R.); mperez@ifc.unam.mx (M.P.d.l.M.); 4 Molecular Neurobiology Laboratory, Department of Biochemistry and Molecular Biomedicine, Universitat de Barcelona, 08007 Barcelona, Spain; catalinaperez@ub.edu; 5 Department of Life Sciences and Biotechnology, Section of Medicinal and Health Products University of Ferrara, 44121 Ferrara, Italy; frl@unife.it (L.F.); rfranco@ub.edu (R.F.)

**Keywords:** G-protein-coupled receptors, oligomerization, heteroreceptor complexes, protein modulation, meta modulation, protein modulation, signal integration, alpha-synuclein

## Abstract

Bioluminescence and fluorescence resonance energy transfer (BRET and FRET) together with the proximity ligation method revealed the existence of G-protein-coupled receptors, Ionotropic and Receptor tyrosine kinase heterocomplexes, e.g., A2AR–D2R, GABAA–D5R, and FGFR1–5-HT1AR heterocomplexes. Molecular integration takes place through allosteric receptor–receptor interactions in heteroreceptor complexes of synaptic and extra-synaptic regions. It involves the modulation of receptor protomer recognition, signaling and trafficking, as well as the modulation of behavioral responses. Allosteric receptor–receptor interactions in hetero-complexes give rise to concepts like meta-modulation and protein modulation. The introduction of receptor–receptor interactions was the origin of the concept of meta-modulation provided by Katz and Edwards in 1999, which stood for the fine-tuning or modulation of nerve cell transmission. In 2000–2010, Ribeiro and Sebastiao, based on a series of papers, provided strong support for their view that adenosine can meta-modulate (fine-tune) synaptic transmission through adenosine receptors. However, another term should also be considered: protein modulation, which is the key feature of allosteric receptor–receptor interactions leading to learning and consolidation by novel adapter proteins to memory. Finally, it must be underlined that allosteric receptor–receptor interactions and their involvement both in brain disease and its treatment are of high interest. Their pathophysiological relevance has been obtained, especially for major depressive disorder, cocaine use disorder, and Parkinson’s disease.

## 1. Introduction

In the early 1980s, the first indications were obtained for the existence of intramembrane receptor–receptor interactions in the central nervous system (CNS) based on the ability of neuropeptides (substance P, neurotensin, chemokine CCK8 and CCK4) to alter the recognition of monoamine receptors in biochemical radio-ligand studies in membrane preparations from the CNS [1]. These results indicated the possible existence of direct physical interactions of different types of receptors in the plasma membrane. Ten years later, it was proposed that the molecular mechanism involved was represented by the existence of different types of G-protein-coupled receptors (GPCRs) heterodimers in balance with GPCR homodimers in the plasma membrane with the potential existence also of higher-order heteromers [2]. A novel molecular integration had been obtained in the plasma membrane, and these heterocomplexes became novel targets for the treatment of neurological and mental diseases [3,4]. In the beginning, the indications obtained were mainly based on studies on GPCRs [5].

Advancements in understanding receptor heterodimerization accelerated with pivotal findings such as the identification of the functional gamma-aminobutyric acid (GABA) B receptor heterodimer [6]. A novel subtype of the GABA B receptor was found, named GABABR2, which formed a receptor interface with GABAR1 based on the yeast two hybrid system [7]. This heterodimer represented the functional GABA B receptor with a receptor interface of a coiled-coil domain. Receptors of the family of taste receptors type 1 (TAS1R) are also a prominent example of GPCR dimerization as they act as obligate functional heteromers: TAS1R1 and TAS1R3 combine to form an umami taste receptor, while the combination of TAS1R2 and TAS1R3 is a sweet taste receptor [8]. While TAS1R taste receptors are known to form functional heteromers, recent evidence suggests that TAS2Rs, which mediate responses to bitter compounds, can also form both homomeric and heteromeric receptor complexes [8,9].

GPCR heteroreceptor complexes may also involve ion channel receptors [10], receptor tyrosine kinases (RTKs) [11], sets of G-protein interacting proteins, receptor activity-modifying proteins (RAMPs) [12] and/or transmitter transporters [13]. The allosteric interactions in such dynamic higher-order receptor complexes occur in an orchestrated spatiotemporal fashion, participating in learning and the formation of molecular engrams for short and long-term memory [3]. In 2000, Fang Liu et al. were the first to demonstrate that GPCR–ionotropic receptor heterocomplexes also exist [14]. Physical protein–protein coupling was observed between dopamine D5R and GABA A receptor. The second intracellular loop of GABA A gamma 2 (short) receptor subunit interacted with the dopamine D5 receptor (D5R) carboxy-terminal domain. Dynamic modulation of synaptic inhibition of the GABA A ion channels was found through inhibitory allosteric receptor–receptor interactions [14]. There exist indications from 1997 that activation of GABA A in membrane preparations reduces the affinity of the high affinity of the dopamine D2 receptor (D2R) agonist binding sites [15] but these results should be validated. It opens the possibility of potential D2R–GABA A interactions. Of special relevance for structural plasticity, for example, in the dendritic tree and its spines, may be the recruitment of RTK to GPCR–RTK heteroreceptor complexes formed, which may result, for example, in synergistic increases in neurite densities and their protrusions in primary neuronal cultures [16,17].

These discoveries underscore the dynamic nature of synaptic excitation/inhibition regulation and hint at the complexity of receptor interactions within the CNS. Allosteric receptor–receptor interactions within GPCR homo- and heteroreceptor complexes further expand the conceptual framework of brain integration and neuropsychopharmacology [18]. These interactions, facilitated by receptor oligomerization, lead to novel receptor dynamics, altering receptor recognition, pharmacology, signaling, and trafficking and potentially giving rise to new allosteric binding sites [19]. Alongside phosphorylation mechanisms, these interactions contribute to fine-tuning receptor function and signaling, providing a basis for diverse physiological responses and potential therapeutic targets. The concept of biased GPCR agonism, which suggests the stabilization of distinct active receptor states leading to selective signaling pathway activation, underscores the importance of receptor diversity and specificity [20,21,22]. The GPCR heterodimer network (GPCR-HetNet) exemplifies how allosteric receptor–receptor interactions amplify GPCR diversity, enhancing signaling specificity and potentially offering novel avenues for drug development in CNS disorders [23]. Moreover, dysfunction within GPCR heteroreceptor complexes may contribute to the pathophysiology of brain diseases, emphasizing the significance of understanding their role in normal brain function [4,24,25].

## 2. On the Existence of GPCR Homo- and Heteroreceptor Complexes

GPCRs exhibit a diverse range of structural characteristics across their phylogenetic families [26]. Family A, known as the rhodopsin-like family, represents the largest subgroup encompassing receptors for odorants, catecholamines, amines, peptides, and glycoprotein hormones. These receptors exhibit highly conserved amino acids, a disulfide bridge linking the first and second extracellular loops (ECLs), and often a palmitoylated cysteine in the carboxy-terminal tail. In contrast, Family B receptors feature a relatively long amino terminus with cysteines forming disulfide bridges but lack sequence homology with Family A. Hormones such as glucagon and parathyroid hormone serve as ligands for Family B receptors. Family C comprises metabotropic glutamate, Ca^2+^-sensing, and γ-aminobutyric acid (GABA)B receptors, characterized by a long amino terminus housing a ligand-binding domain resembling a ‘Venus flytrap’. Unlike Families A and B, Family C receptors lack certain key structural features but possess short and conserved third intracellular loops. Despite their structural disparities, a crucial shared characteristic among all GPCRs is their ability to interact and oligomerize [5,27,28,29]. The structural organization of GPCRs presents a complex and dynamic landscape, with evidence suggesting both dimerization and higher-order oligomerization [5,23].

Over the past two decades, dimerization and oligomerization have been observed for nearly all tested GPCR subtypes (239 GPCR protomers, representing approximately 48% of the total number of 519 true non-orphan human GPCR) in both heterologous systems and native tissue [23,30]. The oligomeric status of GPCRs indeed significantly influences receptor activation and function, but it exhibits variability among different receptors and can change at different stages of the receptor’s life cycle [5]. While class C GPCRs typically require dimerization to transduce transmembrane signaling in response to agonists, class A and B GPCRs can activate G proteins and recruit β-arrestins as monomers upon agonist binding [28,31]. Nevertheless, most tested GPCRs form dimers and oligomers, leading to a spectrum of functional consequences. Functional interactions between binding sites in heterodimeric receptors have been observed in various experiments, opening avenues for novel pharmacology and revealing synergistic or antagonistic effects on signaling [32,33,34].

Additionally, oligomerization provides an additional criterion for endoplasmic reticulum quality control and facilitates cell-surface trafficking [35]. GABAB receptor heterodimerization serves roles beyond cell-surface trafficking [36], affecting mechanisms of GABA binding and receptor-G-protein coupling. Notably, differences in the N-terminus between GB2 and GB1 suggest distinct roles in ligand binding and receptor-G-protein coupling. This suggests a form of trans-activation wherein GB1 binds agonist while GB2 couples to G-protein [37,38]. Similar trans-activation mechanisms have been observed in the luteinizing hormone (LH) receptor, a Family A receptor, indicating a broader role for this mechanism beyond GABAB receptors [39,40]. However, it is essential to note that the stability and stoichiometry of GPCR complexes can vary considerably among Family A and B GPCRs, with cis-activation being the most common mechanism, while only class C GPCRs tend to form stable complexes with trans-activation mechanisms.

Plasma membrane homo- and heteroreceptor complexes were mainly identified with coimmunoprecipitation [28], bioluminescence and fluorescence resonance energy transfer (BRET and FRET [41,42,43]), cross-linking, gel filtration, Fluorescence Correlation Spectroscopy [44,45], biomolecular fluorescence complementation, single-molecule microscopy [46,47,48], quantal brightness, and fluorescence fluctuation (SpIDA) [49] primarily using engineered GPCR constructs expressed in heterologous systems. Analyzing GPCR interactions in native environments has posed significant challenges. However, the use of coimmunoprecipitation [17,50,51] and time-resolved Förster resonance energy transfer (FRET) between GPCR ligands has revealed oligomers in native tissues [52,53,54]. It should also be noted that in the period of 2010–2013, it became possible to demonstrate heteroreceptor complexes in the brain at endogenous expression levels of receptors based on a novel technique, in situ proximity ligation assay (PLA), in combination with immunohistochemistry [17,55,56,57]. This important technique (PLA) was developed by Fredriksson, Gullberg, Söderberg and colleagues [58,59]. For recent work on the analysis and quantitation of the GPCR heterocomplexes with in situ PLA (see [57,60,61,62,63,64,65,66,67,68,69,70,71]).

In current procedures, the receptor complexes appear as red blobs with a diameter of 0.5 to 1.5 um and their density in sampled fields is determined using confocal laser microscopy. The assay is performed in combination with a Neuro-ChromPan neuronal marker antibody-Alexa488, giving a green fluorescence to the neurons and enabling the study of the link of the red blobs to the neurons [61,62,63,64,65]. Based on different types of glial markers, the link of the red blobs to glial cells can also be established. Recently, a new method for Boolean analysis at a molecular level has been introduced for protein interactions [59]. The method is called MolBoolean and uses the Boolean operations AND and NOT at the molecular level. Like the PLA method, the pool of protein A and protein B is visualized when they are in sufficient proximity (AND). The difference with the MolBoolean method is that it also visualizes proteins A and B when they do not take part in an interaction with each other (NOT). Thus, the relative quantities of non-interacting protein A and protein B can be determined. It should be considered that the non-interacting components protein A and protein B, at least some of them, interact with other types of proteins or form homomers. This aspect is of high relevance for understanding the complexity and diversity of receptor–receptor interactions.

Quantitative studies aiming to determine the monomeric/oligomeric composition of certain class A and B GPCRs have revealed an equilibrium between monomeric, dimeric and higher-order oligomeric species, suggesting a more complex picture [44,45,47,48,72,73,74,75,76,77,78]. Studies using different approaches have yielded conflicting results regarding the existence and composition of GPCR oligomers, with some suggesting that observed monomers and dimers may represent dissociation products from larger oligomeric complexes [27]. Additionally, limitations in the resolution of certain techniques used to study GPCR oligomerization may fail to capture rapid fluctuations in receptor interactions, while the cellular environment can influence the stability and composition of receptor–receptor interfaces. Evidence indicates that ligand binding can modulate the extent and stability of GPCR interactions, adding another layer of complexity to their quaternary organization [31,79,80]. For example, ligand binding to the dopamine D2 receptor (D2R) stabilizes dimers [74], while the muscarinic acetylcholine M2 receptor (mAChR2) appears to predominantly exist as a monomer but can reversibly form dimers at the plasma membrane under certain conditions [81]. However, studies employing fluorescence correlation spectroscopy (FCS) with photon counting histogram analysis to investigate the oligomer status of six class A GPCRs (serotonin 5-HT2A, alpha-1B adrenergic receptor, beta-2B adrenergic receptor), mAchR1, mAchR2 and dopamine D1R) indicated that these receptors predominantly exist as homodimers within the plasma membrane, with no evidence of tetramers or higher-order oligomers, suggesting a stable configuration unaffected by agonist treatment or receptor expression levels [44]. Frizzled 6 (FZD6), a Class F GPCR involved in WNT protein signaling, has been shown to form dimers regulated by WNT proteins [82]. Live cell imaging techniques revealed that agonist-induced dissociation/re-association of FZD6 dimers is crucial for signaling to extracellular signal-regulated kinases1/2. This discovery of agonist-dependent dynamics of dimers extends our understanding of Class F and other dimerizing GPCRs, presenting novel targets for therapeutic intervention.

An implicit assumption in the ongoing debate about monomer versus dimer status is that the quaternary structure and functional state of all GPCRs remain constant throughout their lifecycle. However, this assumption overlooks the dynamic nature of GPCR behavior from synthesis in the endoplasmic reticulum to internalization and sorting in endosomes. Each GPCR subtype exhibits a unique combination of structural and functional features, including variations in N-termini, cytoplasmic loops and C-termini, as well as differing requirements for phosphorylation and interactions with downstream signaling molecules and lipids, which may be prominent driving factors for oligomerization [83,84,85]. Nevertheless, discrepancies in the quaternary structure and stability of GPCR complexes across different studies underscore the need for further refinement and systematic comparison of methods to monitor GPCR interactions over time.

The remarkable structural and functional diversity of the GPCR superfamily, acquired over billions of years of evolution, suggests that each receptor may function differently in terms of oligomerization state and activity. Furthermore, the efficiency and specificity of GPCR signaling have prompted the suggestion that GPCRs may signal within discrete nanodomains on the plasma membrane or form stable complexes with G proteins and effectors [86,87]. Recent studies utilizing innovative optical methods like FRET [52,54], single-molecule microscopy [46,48,49,75] and in situ PLA [56,57,68] have begun to delve into the organization of GPCR complexes and signaling in living cells on spatial and temporal scales.

Initially, many experiments focused on the binary characterization of interactions between GPCRs, perhaps partly due to technical limitations. However, after compiling a vast amount of experimental evidence, we proposed the broader concept of considering GPCRs as homo- and heteroreceptor complexes. We also emphasized the need to consider the relevance of the balance between the different complexes’ populations within the membrane of cells or nanodomains [88]. These studies reveal a complex and highly dynamic picture wherein GPCRs transiently or stable interact with other membrane proteins/receptors (such as involve ion channel receptors, receptor tyrosine kinases, receptor activity-modifying proteins and/or transmitter transporters), their signaling partners (G proteins, B-arrestin), membrane lipids, and the cytoskeleton to form long-lasting or short-lived receptor complexes and signaling nanodomains both on the plasma membrane and intracellularly.

### 2.1. GPCR-GPCR Heterocomplexes

As already indicated, the work on neuropeptide-monoamine receptor–receptor interactions in the CNS indicated the existence not only of GPCR monomers [89] but also of GPCR homo and heteroreceptor complexes [5,90,91]. It can include receptor dimers, higher-order receptor complexes and receptor-interacting proteins like different types of adapter proteins and synaptic and/or non-synaptic proteins [18,85,92].

The GPCR heterodimer network (www.gpcr-hetnet.com, last update 2014 [23], accessed on 23 April 2024) provides insight into the direct interactions between GPCRs, revealing a scale-free model where a few protomers dominate connectivity (e.g., adenosine A2A receptor, dopamine D2R and B2-adrenergic receptor). Experimentally verified interactions were reported for 156 GPCR protomers, representing approximately 20% of the total putative human GPCR protomers (a total number of 797 human GPCR exists, including around 300 orphan receptors). Notably, interactions were most incomplete for the rhodopsin-like superfamily despite representing the majority of identified protomers, even though they represented 18–25% of the interactions. The Secretin-like superfamily and metabotropic glutamate receptor-like superfamily exhibited higher interaction rates, with 33% and 60% of putative protomers involved in interactions, respectively. While more than 87% of identified protomers exist as homomers, the balance between homo- and heteromer populations is crucial, potentially influencing pathological diseases where GPCR dimerization plays a role. Intrafamily connections were significantly more prevalent than interfamily connections, possibly due to favorable co-evolution of protomer interfaces within subfamilies and diverse cell and tissue expression patterns. Further research into GPCR heterocomplexes specificities may reveal cross-family heterodimerization or intrafamily specificities, shedding light on the complex landscape of GPCR interactions.

For drug development in CNS disease, it is of particular interest to understand the interface of the GPCR dimers. In 2004, it became possible to demonstrate with mass spectrometry and pulldown techniques to begin to understand the receptor interface based on the findings of direct epitope–epitope electrostatic interactions between the A2AR–D2R protomers involving the third intracellular loop of the D2R and the C-terminal tail of the A2R [93]. In 2010, it was demonstrated by Borroto-Escuela et al. [94] that a serine point mutation in the C-terminal tail of the A2AR diminished the heteromerization and also found for the first time that the transmembrane helices were involved.

In 2018, a structural model of the A2AR–D2R heterodimer was obtained by mapping its interface [95]. A computational and experimental study was performed. The modeling of the regions of the receptor interface was performed by means of peptides from the transmembrane helices and their effects on the A2AR–D2R using BRET and PLA and modulation of the D2R binding. Peptides belonging to TM-IV and TM-V of the A2AR counteracted the heteromer formation and antagonized the A2AR agonist-induced allosteric inhibition of the affinity of the D2R. Protein–protein docking allowed us to provide a model of the A2AR–D2R containing the TM-IV and TM-V interface. The model was improved by molecular dynamic simulation. Mutations in this receptor interface reduced the allosteric inhibition of the D2R protomer and brought down the BRET signal. The results of this approach suggest that it will be useful for building models of other GPCR heterocomplexes. In this way, the receptor interface of GPCR heterocomplexes can be characterized, which will assist in the development of novel drugs for the treatment of neurological and mental diseases.

One-third of the approximately 400 nonodorant GPCRs remain orphans, with unidentified ligands and potential ligand-independent functions [96,97]. Members of the GPCR family often modulate other receptors through heterodimerization. For instance, GPR50, an orphan GPCR, interacts with the melatonin MT1 receptor, influencing their signaling pathways [98]. Similarly, the orphan receptor GPR143 interacts with dopamine receptors D2R and D3R, suggesting implications for neurological conditions such as Parkinson’s disease [97]. GPR18 and GPR55, orphan receptors, heterodimerize with cannabinoid CB1 and/or CB2 receptors, exhibiting negative cross-talk and bidirectional cross-antagonism, suggesting their involvement in neurodegenerative diseases like Alzheimer’s and Parkinson’s [70,71,99,100].

Oligomerization of GPCRs is not exclusive to the CNS, where multiple GPCR subtypes are often expressed within the same neuron or glial cells but is also observed in peripheral tissues. This process is fundamental for fine-tuning cellular responses and coordinating various physiological processes, including reproductive functions, immune system regulation, and cardiovascular homeostasis. In the periphery, both homo- and heteroreceptor complexes of GPCRs are prevalent and play crucial roles in regulating physiological functions. For instance, follicle-stimulating hormone (FSHR) and luteinizing hormone/chorionic gonadotropin (LHCGR) receptors form homodimers and heterodimers, which are essential for folliculogenesis, the process of ovarian follicle development [101]. The activation of these receptor complexes initiates signaling cascades crucial for follicle maturation, ovulation, and subsequent reproductive processes [101]. Moreover, the heterodimerization of angiotensin II type 1 receptor (AT1R) with the bradykinin B2 receptor (B2R) influences cardiovascular regulation, including blood pressure control and vascular tone modulation [102,103]. Additionally, the formation of chemokine receptor heterodimers, such as CXCR4 and CCR5, impacts immune cell migration and inflammatory responses [104,105].

### 2.2. GPCR-Ion Channels Heterocomplexes

The interactions between ion channels and GPCRs play pivotal roles in cellular signaling and physiological processes. It was demonstrated by Lee et al. (2002) that the NR1 and NR2 subunits of the NMDAR formed a heterodimer with the D1R [10]. It involved two areas of the D1R carboxyl tail and caused a reduction of the NMDAR currents, which brought down excitotoxicity. Currently, interfering peptides are being used to disrupt its operation to study the function of the D1R-NR1 complex [106]. Furthermore, in 2006, the NR2B subunit of the NMDAR was shown to form a heterodimer with the D2R [107]. Their interactions were modulated in response to cocaine, indicating their possible relevance for understanding the actions of cocaine. It has been observed an interaction between neurotensin receptor 1 (NTS1) and NMDA R [108]. Perroy et al. demonstrated a direct physical interaction between mGlu5a and NMDA receptors, leading to reciprocal inhibition of their respective functions. This interaction, observed in hippocampal neurons, implies a higher degree of target-effector specificity and subcellular signaling localization than previously understood. The deletion of the C terminus of mGlu5a abolished this interaction, highlighting the importance of this region in mediating the functional cross-talk between these receptors.

Furthermore, Marino et al. [109] showed that muscarinic M1 receptors (M1Rs) potentiate NMDA receptor currents in hippocampal pyramidal cells, suggesting a role in learning and memory processes. They demonstrated colocalization of M1Rs and NR1a NMDA receptor subunits, indicating a spatial relationship that allows for physiological interactions between these receptors. This finding has implications for understanding neurodegenerative diseases like Alzheimer’s [109].

A study by Liu et al. [14] uncovered a selective complex formation between GABA(A) ligand-gated channels and D5 receptors. This interaction occurs through direct binding between the D5 receptor carboxy-terminal domain and the second intracellular loop of the GABA(A) gamma2 (short) receptor subunit. This association facilitates mutually inhibitory functional interactions between these receptor systems, suggesting a previously unknown mechanism for regulating synaptic strength and potential implications for psychomotor disease states.

Additionally, voltage-gated calcium channels, crucial regulators of calcium homeostasis, are finely tuned by cellular signaling pathways, including those activated by GPCRs. GPCRs not only regulate calcium channel activity via second messengers but can also physically associate with calcium channels to directly influence their functions and trafficking [110,111]. Furthermore, specific populations of ion channels are directly controlled by G proteins, while others are modulated indirectly through G-protein-dependent phosphorylation events and lipid metabolism. These diverse modifications affect ion channel activities and spatiotemporally regulate membrane potentials and intracellular calcium concentrations. Moreover, the family of G-protein-gated inwardly rectifying potassium channels (Kir3 or GIRK) expressed in the brain, heart, and endocrine tissues were recently shown to stably associate with several different GPCRs, forming the basis of a macromolecular ion channel–GPCR signaling complex [112,113,114]. The molecular determinants that mediate and maintain GPCR-GIRK channel complexes are currently not well understood; however, these protein–protein interaction processes are crucial in determining both the synaptic response times and the extent of GPCR “crosstalk” in GIRK-mediated inhibitory synaptic transmission [113].

The interactions between nicotinic acetylcholine receptors (nAChRs) and dopamine receptors are another example of GPCR–ion channel heterocomplexes, which reveal complex mechanisms underlying synaptic modulation and neuronal excitability [115]. nAChRs are ligand-gated cationic channels composed of various α and β subunits, existing as α7-containing (α7nAChRs) and non-α7 nAChRs. Dopamine receptors, including D2 dopamine receptors (D2ARs), co-localize with nAChRs in dopamine (DA) neurons within the ventral tegmental area (VTA) and substantia nigra (SN), as observed in soma, axons, terminals, and other neuron types. Specifically, α6-containing nAChRs are highly expressed in midbrain DA neurons, where their activation increases neuron firing, a process antagonized by D2ARs. Quarta et al. [116] demonstrated that nicotine-induced dopamine release in the striatum is modulated by D2 autoreceptors and non-α7 nAChRs. Co-immunoprecipitation experiments revealed physical interactions between β2 subunits of non-α7 nAChRs and D2 autoreceptors, suggesting the formation of heteromeric dopamine autoreceptor complexes that modulate dopamine release. These findings highlight a potent crosstalk between G-protein-coupled receptors and ligand-gated ion channels in dopaminergic nerve terminals. Also, the activation of nAChRs induces morphological remodeling in DA neurons, a process dependent on functional DA D3 receptors (D3Rs). Evidence suggests the existence of D3R-nAChR heteromers [117], with direct interaction between D3R and the β2 subunit of nAChR. Disruption of these heteromers by interfering peptides targeting intracellular loops reduces nicotine-induced neurotrophic effects on DA neurons, emphasizing the functional significance of the D3R-nAChR heteromer in mediating nicotine’s effects.

### 2.3. GPCR–RTK Heterocomplexes

Flajolet et al. [16] discovered that fibroblast growth factor receptor 1 (FGFR1) can form heteroreceptor complexes with A2AR, a GPCR., based on the yeast two-hybrid method. Coactivation of the two protomers resulted in neurite extension of the cells and enhanced cortico-striatal plasticity. Twelve years later, it was found that A2AR also formed heteroreceptor complexes with tropomyosin receptor kinase B (TrkB) receptors in the dorsal hippocampus using in situ PLA [118]. The complexes were inter alia found in high densities in the pyramidal cell layers of the CA1–CA3 regions but lacked presence in the molecular and granular cell layers of the dentate gyrus. These A2AR–TrkB heteroreceptor complexes may have implications for hippocampal plasticity, which is impaired in aging [118].

The discovery of the FGFR1–5-HT1A heteroreceptor complexes in the dorsal hippocampus was made in 2012 [17] using PLA, followed by observations of their presence in the dorsal raphe. In the dorsal raphe, the FGFR1 forms a complex with the 5-HT1A auto-receptor. It was found that combined 5-HT1AR agonist and FGF2 treatment increased the density of these heteroreceptor complexes in the hippocampus. Furthermore, the enhanced positive allosteric receptor–receptor interactions in these complexes led to improved FGFR1 signaling linked to antidepressant actions [11]. Taken together, these results bring together the serotonin and neurotrophic hypothesis of major depression.

Disturbances have been observed in the FGFR1–5-HT1AR heterocomplexes in the raphe-hippocampal 5-HT neuronal system in a genetic rat model of depression (Flinders sensitive line rat) [25]. Such deficits may involve a failure of combined agonist treatment to uncouple the 5-HT1A auto receptor from the GIRK channels in the raphe 5-HT nerve cells, which increases their hyperpolarization and may reduce their firing. This may be related to a reduced ability of the FGFR1 protomer to reduce the signaling of the 5-HT1A auto-receptor protomer via allosteric receptor–receptor interactions [25]. A neurochemical and electrophysiological analysis demonstrated that astrocytic FGFR1–5-HT1AR heterocomplexes also exist in the hippocampus [119]. Localization of hippocampal FGFR1–5-HT1AR heterocomplexes in astrocytes was found using in situ proximity ligation assay combined with immunohistochemistry using glial fibrillary acidic protein (GFAP) immunoreactivity as a marker for astroglia. Acute i.c.v. treatment with 8-OH-DPAT alone or together with basic fibroblast growth factor (FGF2) significantly increased FGFR1–5-HT1AR heterocomplexes in the GFAP positive cells, especially in the polymorphic layer of the dentate gyrus (PoDG), but also in the CA3 area upon combined treatment. Also, structural plasticity changes were observed in the astrocytes, especially in the PoDG region, upon these pharmacological treatments [119]. FGFR1–5-HT1AR heterocomplexes in astrocytes modulate the structure and function of astroglia in the hippocampus, leading to possible changes in the gamma oscillations.

There also exist indications for the existence of muscarinic acetylcholine receptor, mAChR–FGFR1 heteroreceptor complexes, which should be linked to the cholinergic neurons [120], which is of high interest. It was associated with the enhancement of neuritis outgrowth in neural hippocampal cultures.

Our recent report on the complex network of interactions between different GPCR–RTK pairs, comprising around 181 GPCR–RTK receptor–receptor interactions (https://www.gpcr-hetnet.com/, accessed on 23 April 2024), highlights the extensive crosstalk and signal integration occurring between these signaling pathways. This GPCR–RTK heteroreceptor complexes diversity sheds light on the intricate ways in which cells coordinate responses to various stimuli, offering valuable insights into the complexities of cellular signaling. Such findings could have significant implications for drug development and therapeutic strategies aimed at modulating GPCR–RTK interactions to treat various diseases and disorders.

## 3. Molecular Integrations through Allosteric Receptor–Receptor Interactions in Heteroreceptor Complexes of Synaptic and Extra-Synaptic Regions

### 3.1. From Modulation of Receptor Protomer Recognition, Signaling and Trafficking to Functions in the CNS, including Behavioral Studies

Early on there have been many studies on the existence of allosteric receptor–receptor interactions in brain dopamine (DA) transmission belonging to the basal ganglia. It is based on biochemical binding studies [121], especially with regard to A2AR–D2R interactions but also to A2AR–D3R complexes [122] and the potential existence of A2AR–D4R complexes [123]. The dopamine (DA) receptors are mainly located in extra-synaptic regions, which is likely also true for these heterocomplexes. In 1998, antagonistic A1R–D1R interactions were also observed in binding studies in the basal ganglia, likely reflecting allosteric receptor–receptor interactions in the extra-synaptic regions. Later, in 2003 and 2008 [124], studies on BRET and FRET on the existence of A2AR–D2R and A1R–D1R heterocomplexes with the A2AR–D2R complexes mainly modulating the striatal-pallidal GABA neurons, known to inhibit the initiation of movements and the A1R–D1R complexes mainly modulating the direct GABA pathway, known to enhance movements. The DA receptor subtypes also interact with each other to form, e.g., D2R–D4R [55] and D1R–D2R [125] heteroreceptor complexes, especially in the basal ganglia. The available evidence suggests that the allosteric receptor–receptor interactions can involve bi-directional modulation of both receptor recognition, signaling and trafficking [123], and include high-order heteroreceptor complexes.

Furthermore, the richness of 5-HT receptor subtypes in the brain is well-known [126], and they form a large number of 5-HT heteroreceptor complexes among themselves like 5-HT1AR–5-HT2AR heterocomplexes [34] and with other types of receptors like the DA receptor subtypes. The DA and serotonin nerve terminal networks are also known to overlap with each other in multiple brain regions. It is, therefore, of substantial interest that D2R–5-HT2AR and D2R–5-HT1A heteroreceptor complexes have been identified in the brain [127,128]. And that only the hallucinogenic 5-HT2AR agonists could enhance the Bmax values and the affinity of the high-affinity component of the D2R protomers through allosteric receptor–receptor interactions in the dorsal and ventral striatum with the D2R signaling also becoming increased [129]. One molecular mechanism for the ability of atypical antipsychotic drugs to diminish psychosis can, therefore, be by blocking the allosteric enhancement of D2R protomer signaling through, e.g., 5-HT2AR antagonism.

It should also be considered that the 5-HT2AR forms a heterocomplex with the oxytocin receptor (OXTR), but in this receptor complex, it exerts an allosteric antagonistic action on the oxytocin receptor signaling [65]. In view of the importance of oxytocin for social behavior and for reward, this action of the 5-HT2AR agonist will contribute to its known depressant actions [24]. The OXTR represents a key hub in the GPCR heteroreceptor network with significant relevance for brain and behavior, and even stronger antagonistic allosteric actions are exerted on the oxytocin receptor protomer by the 5-HT2CR protomer in OXTR–5-HT2CR heterocomplexes [64].

We should also consider the DA and serotonin heteroreceptor complexes as key hubs in the integration of DA and serotonin transmission [23]. It is of substantial interest that D2R–5-HT1AR heterocomplexes also have been discovered [130]. The method was based on the FRET principle and fluorescence lifetime imaging microscopy. These complexes were found to a high degree in the medial prefrontal cortex while found to a much lower extent in the striatum. In 2018, it was found that the atypical antipsychotic drug risperidone in a low dose which can reduce both D2R and 5-HT1AR protomer signaling, increased the D2R–5-HT1AR heteromerization, using in situ PLA, in the prefrontal cortex of the mouse [128]. It may reflect an enhancement in the affinity of the two receptor protomers for each other in the prefrontal cortex that may, e.g., lead to enhanced inhibition of the D2R and /or 5-HT1AR protomer signaling, which should be further investigated.

There also exist 5-HT1AR–5-HT2A heteroreceptor complexes in the anterior cingulate cortex and the hippocampus [34]. It should therefore be tested if also higher-order D2R–5-HT1A–5-HT2A exist in a dynamic balance with D2R–5-HT1A and D2R–5-HT2AR heterocomplexes, also including the corresponding homo-, isomeric complexes and monomers. These results underline the fundamental role the various DAR–5-HTR heterocomplexes can have in the integration of the DA and 5-HT signaling in the dopamine and serotonin nerve terminal networks [131].

### 3.2. Expanding the Concept of Meta-Modulation (Second-Order Modulation) and Protein Modulation Based on the Existence of Allosteric Receptor–Receptor Interactions in Heteroreceptor Complexes

The modulation of volume transmission [132,133,134,135] was described by Agnati et al. [136] to show multiple ways to modulate volume transmission through metabolic signals, temperature gradients and pressure waves. In a book on “Beyond Neurotransmission” from 1999, edited by Paul Katz, the term meta-modulation was given to “the control and modulation of neuromodulation [137]. Furthermore, the term “meta-plasticity” was introduced in this book to describe the “plasticity of synaptic plasticity” [138].

**Figure 1:**
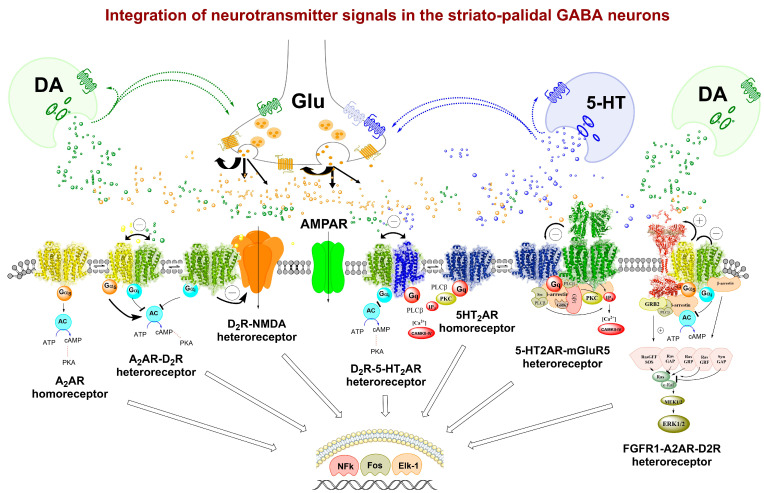
Functional Interaction of GPCR Heteroreceptor Complexes in striato-palidal GABA neurons. This figure illustrates the intricate functional interplay and roles of GPCR homo- and heterocomplexes within GABAergic neurons, including those involving receptor tyrosine kinases (RTKs) and ion channels. The balance between these complexes and their allosteric receptor–receptor interactions is depicted, with the nature of these interactions indicated in the top part of each receptor complex. Antagonistic allosteric modulation is denoted as (−), while facilitatory allosteric modulation is represented as (+). Dopamine D2R heteroreceptor complexes are proposed to primarily localize in extrasynaptic regions but may also be found in synaptic locations. These complexes are suggested to modulate synaptic glutamate transmission in striato-palidal GABA neurons. Additionally, the potential existence of NMDAR–D2R heterocomplexes in striato-palidal GABA neurons could contribute to the reduction of glutamate drive through D2R protomer-mediated inhibition of NMDA receptors. These molecular integrative receptor mechanisms operate both in extra- and presynaptic locations, significantly modulating neurotransmitter release. Integration among multiple heteroreceptor complexes within extra- and postsynaptic sites leads to notable alterations in recognition, signaling, and trafficking. The concept of meta-modulation, introduced in 1999, describes the fine-tuning or modulation of nerve cell transmission through receptors of different types located within the same nerve cell and exhibiting functional interactions. This depiction extends the concept of meta-modulation (second-order modulation) based on the existence of physical receptor–receptor interactions in heteroreceptor complexes and their allosteric receptor–receptor interactions within synaptic and extrasynaptic membranes. The following depicted GPCR homo- and heteroreceptor complexes are illustrated: adenosine A2AR [73], dopamine D2R [74] and serotonin 5HT2AR [44,148] homoreceptor complexes, A2AR–D2R [149,150], D2R–5HT2AR [127], 5HT2AR–mGluR5 [151], D2R-NMDA [107] heteroreceptor complexes and the putative FGFR1A–A2AR–D2R heteroreceptor complexes [11,16,41]. Other glutamate receptors, such as α-amino-3-hydroxy-5-methyl-4-isoxazolepropionic acid receptor (AMPA) and kainate receptors, may also be present on striato-palidal GABA neurons, playing crucial roles in enhancing glutamate activation, thereby increasing glutamate drive and activity in mesolimbic DA reward neurons.

It should be noted that the discovery of GPCR receptor–receptor interactions in the plasma membrane was made already in the early 1980ies with the hypothesis in 1993 that these receptor–receptor interactions were caused by the formation of homo and hetero receptor dimers and higher-order homo- and heteroreceptor complexes [2]. Thus, it seems clear that the concept of meta-modulation builds on the existence of physical receptor–receptor interactions in heteroreceptor complexes in synaptic and extra-synaptic membranes [10,23] (Figure 1). The molecular integrative receptor mechanisms are in operation both in extra-and presynaptic and extra-and postsynaptic locations. They play a major role in modulating pre -and extra-synaptic release of neurotransmitters [134,135]. In the extra-and postsynaptic places, the integration in and between multiple heteroreceptor complexes will lead to significant alterations in recognition, signaling and trafficking of the extra-and postsynaptic heteroreceptor complexes. The introduction of receptor–receptor interactions [139] was the origin of the concept of meta-modulation provided by Katz and Edwards [137]. Meta-modulation in 1999 stood for the fine-tuning or modulation of nerve cell transmission through receptors of different types located in the same nerve cell and with functional interactions.

Ribeiro and Sebastiao, based on a series of papers, provided strong support for their view that adenosine can meta-modulate (fine-tune) synaptic transmission through adenosine receptors [140,141,142]. They also cite the work of Fields and Burnstock [143] on the relevance of ATP and adenosine as fine-tune modulators since these authors underline the role of ATP and adenosine in glia–neuron cross-talk. Based on the existence of multiple A2AR and A1R heteroreceptor complexes in the brain [144,145], the mechanism for the ability of adenosine to fine-tune or meta-modulate synaptic and extra-synaptic complexes lies in different types of adenosine heteroreceptor complexes formed in various brain circuits [146,147]. The formation of heteroreceptor complexes is, in fact, a general integrative CNS mechanism involving, among others, GPCR, RTK and ionotropic receptors [18,23]. The physical receptor–receptor interactions make possible the allosteric receptor–receptor interactions with fine-tuning of the participating receptor protomers in terms of recognition, signaling and trafficking as well as transmitter release [88]. It is an essential integrative mechanism that plays a major role in meta-modulation by modulating presynaptic and extra-synaptic transmitter release and postsynaptic and extra-synaptic neuronal activity, including firing.

Ribeiro and Sebastiao [140] have discussed the fine-tuning of interactions between A2AR and calcitonin gene-related peptide (CGRP), A2AR and α7nAChRs and A2AR and TrkB interactions. The fine-tuning of these interactions can reflect the formation of heteroreceptor complexes with direct allosteric receptor–receptor interactions [118,146,147]. The A2AR can also potentially directly interact with the adenosine transporter and GABA transporter proteins, leading to the enhancement of adenosine and GABA transport [140].

Not only adenosine receptor subtypes but also, e.g., serotonin and dopamine receptors, representing GPCRs, participate in many heteroreceptor complexes, while other types of GPCRs can have low participation in forming heterocomplexes (Figure 1). GPCRs also form heterocomplexes with ionotropic receptors like NMDAR and AMPAR and RTK like TrkB and FGFR1. They perform the integration of signals both in synaptic and volume transmission [134]. The term meta-modulation can be used to describe integration through direct allosteric receptor–receptor interactions in heterocomplexes, including presynaptic and postsynaptic regions, as well as extra-synaptic regions [134,152]. It can involve e.g., presynaptic release-regulating metabotropic glutamate receptors [153].

Meta-modulation mechanisms have also been described in relation to release-regulating presynaptic receptors based on the use of synaptosomes that lack the post-synaptic connection [154]. However, in the future, it will be important to develop techniques to understand the link between the pattern of presynaptic transmitter release and the pattern of activation and inhibition of the postsynaptic receptors and their allosteric receptor–receptor interactions, a process which can be best described as receptor reorganization through protein modulation.

The existence of heteroreceptor complexes in the presynaptic and extra-synaptic nerve terminal regions should also be considered to fine-tune through protein modulation of receptors and transporters, the transmitter release from the presynaptic and extra-synaptic terminal regions, often forming varicosities. Together with signals from the postsynaptic surface, the pattern of neurotransmitter release from the synaptic terminal can reach the state required to mediate the firing pattern that should be learned through the reorganization of multiple heteroreceptor complexes on the postsynaptic side. In this way, learning is accomplished [155].

The work of Pittaluga et al. [156] gives indications that the neuropeptide somatostatin modulates presynaptic glutamate release and signaling that may involve somatostatin receptor-glutamate receptor interactions in the glutamate nerve terminal. It can be associated with protein modulation (fine-tuning) of presynaptic glutamate receptor activity with alterations of glutamate release increasing synaptic strength through the activation of postsynaptic glutamate receptors protomers participating in postsynaptic heteroreceptor complexes.

In addition, it should be underlined that the reorganization of the postsynaptic heteroreceptor complexes with altered synaptic receptor–receptor interactions can represent the molecular basis of learning and their consolidation through the formation of novel adapter proteins that bind to and stabilize these heteroreceptor complexes. These molecular changes can lead to long-term memory with the formation of the molecular engram [3,157,158]. The major role of dynamic synaptic heteroreceptor complexes lies in their ability to accomplish learning through their reorganization via protein modulation. It is followed, if the change is relevant, by the transformation of the new complexes into long-term memories by causing transcription of novel types of adaptor proteins that bind to the reorganized heteroreceptor complexes in the plasma membrane. They are stabilized by linking the modulated receptor complexes together and linking them also to synaptic plasma membrane proteins like cell adhesion proteins and master scaffolding proteins.

In learning and memory, modulated receptor–receptor-protein interactions in high order synaptic heteroreceptor complexes lead to marked changes in their structure and function that can be described as protein modulation. A suitable name for studies on learning and memory at the molecular level would be again protein modulation, which could be a general name to study receptor–receptor interactions and receptor-ligand interactions at the molecular level, also including studies on protein phosphorylation and de-phosphorylation.

## 4. Learning, Memory, and Synchrony at the Molecular Level

A key role is given to multiple pre- and postsynaptic high-order heteroreceptor complexes in learning and memory [3]. The molecular basis of learning and memory can be based on the receptor reorganization, through protein modulation, of the homo and heteroreceptor complexes in the postsynaptic membrane [134,155,158]. It also leads to changes in the presynaptic receptor complexes in order to facilitate the altered pattern of transmitter release to be learned [135]. The temporal pattern of release of transmitters leads to a transient reorganization of the postsynaptic homo and heteroreceptor complexes. It reflects a learning process that can be transformed into a short-term memory. As a result of the homo and heteroreceptor complexes signaling and their impact on gene expression and chromatin reorganization, novel adapter proteins are formed that lead to a consolidated generation of the pre-and postsynaptic heteroreceptor complexes. Such stabilized heteroreceptor complexes become a long-term memory, a so-called molecular engram. It takes place through the stabilization of the synaptic heteroreceptor complexes brought about by multiple novel adapter proteins that bind and link the multiple synaptic heteroreceptor complexes together with each other and postsynaptic proteins, like cell adhesion proteins and master scaffolding proteins, enabling the consolidation.

To understand how the synchrony [159] between neuronal cell populations can be established, it is proposed that the neurons with novel memories can play a role through volume transmission [135], involving their release of exosomes containing the novel types of adapter proteins used for long term memory, can play a role. Such novel adaptor proteins may favor synchrony development upon their internalization by making it possible to form some similar synaptic heteroreceptor complexes as found in the original nerve cell population. Through their original adapter proteins, the possibility of forming similar memories in other neurons may develop via unique adapter proteins containing exosomes that can enter other nerve cells via volume transmission and internalization. Such molecular events leading to the selection of similar heteroreceptor complexes in additional and close-by nerve cells should help the development of synchrony with the improvement of the functional events needed, like the addition of complementary movements and cognitive functions. Complementarily, the novel transcription factors and activator proteins formed in the first nerve cell population can, at least in part via VT through exosomes, also be internalized into the second nerve cell populations to help them reach coordination with the first neural population. A key gene may be triggered by adapter proteins, transcription factors and/or activator proteins to reach such a coordination with a similar neural synchrony as in the first nerve cell population, which allows context to develop between first and second nerve cell populations to develop.

## 5. Allosteric Receptor–Receptor Interactions and Their Involvement in Brain Disease and Its Treatment

### 5.1. Pathophysiological Relevance for Major Depressive Disorder and Potential Drug Development

Certain central 5-HT heteroreceptor complexes in the brain have been indicated to become dysfunctional in models of major depressive disorder and can represent new targets for treatment not only of major depressive disorder but also of anxiety disorder [11,25,160]. Currently, the aim is to identify the most vulnerable 5-HT heteroreceptor complexes. Recent work indicates that the 5-HT1AR–R5-HT2AR [34] and FGFR1–5-HT1AR heterocomplexes [17,25,119] are of special interest. The FGFR1–5-HT1A auto-receptor complex in the dorsal raphe nerve cells should also be mentioned, with the FGFR1 protomer reducing the inhibitory 5-HT1AR auto-receptor protomer function by diminishing the 5-HT1AR auto-receptor agonist-induced opening of the GIRK channel. The 5-HT1A auto-receptor reduces the activity of the raphe-limbic serotonin neurons, which can be enhanced in a genetic rat model of depression [25]. In FSL rats compared to Sprague-Dawley (SD) rats, reductions can develop in the ability of the 5-HT1AR agonist 8-OH-DPAT and combined FGFR1 and 5-HT1AR agonist treatment to increase the density of FGFR1–5-HT1AR heteroreceptor complexes in the dorsal raphe nucleus. It was proposed that such deficits in FSL rats may lead to a failure of such agonist treatments to uncouple the 5-HT1AR auto-receptors from the GIRK channels. Such events may contribute to the failure of producing antidepressant-like effects in the FSL rat with such combined agonist treatment as found in the SD rat. On the other hand, the activation of the serotonin auto-receptor enhances the trophic activity of the FGFR1 protomer, increasing the survival of the dorsal raphe serotonin neurons with improvement of their dendritic and nerve terminal networks [11,17].

It has become increasingly clear that oxytocin and its receptor play a significant role in the emotional networks of the brain, including the limbic regions and the hypothalamus [161,162,163]. It is therefore of high interest that oxytocin receptors can form heteroreceptor complexes with D2R [33,164], 5-H2AR [65] and 5-HT2CR [64] in the central nervous system. Facilitatory allosteric receptor–receptor interactions were observed in the D2R–OXTR complexes, enhancing the functions of the two receptor protomers. This D2R–OXTR complex could therefore be a new target for improving the emotional networks through enhanced oxytocin receptor signaling in the two regions studied, the dorsal and ventral striatum In contrast, the molecular, biochemical and behavioral evidence obtained in the studies on the 5-HT2AR–OXTR and 5-HT2CR–OxytocinR heterocomplexes indicated that especially the 5-HT2AR and 5-HT2CR agonists had a significant and substantial ability to inhibit the Gq signaling of the oxytocin receptor. In view of the ability of 5-HT2AR and 5-HT2CR activation to enhance depressive actions [64,65], it is possible that this mechanism can involve an inhibitory allosteric modulation of the oxytocin receptor protomer signaling.

Other evidence also suggests that there are functional or physical interactions between serotonin (5-HT2AR) receptors and metabotropic receptors (mGluRs), which play significant roles in neuropsychiatric disorders such as schizophrenia and depression [165,166]. The 5-HT2A receptor is known to interact with various metabotropic glutamate receptors (mGluRs), particularly mGluR2 [167,168,169]. Studies have demonstrated the formation of functional complexes between 5-HT2ARs and mGluR2 in the brain cortex [169]. González-Maeso et al. [166] identified specific transmembrane helix domains involved in the interaction between mGluR2 and 5-HT2ARs. Disruption of these complexes has been associated with alterations in cellular signaling and behavioral responses, particularly psychosis-like effects induced by hallucinogenic drugs. Post-mortem brain analysis of untreated schizophrenic subjects revealed dysregulated expression of 5-HT2AR and mGluR2 receptors, suggesting their involvement in schizophrenia pathology [166]. The mGlu5 receptor has also been implicated in interactions with 5-HT2ARs, particularly in the regulation of locomotor activity. Halberstadt et al. [151] demonstrated that loss of mGlu5 receptor activity, either through pharmacological means or gene deletion, led to locomotor hyperactivity in mice. Gene deletion of mGlu5 receptors increased the behavioral response to the 5-HT2AR agonist DOM, suggesting a functional interaction between these receptors in modulating locomotor activity. These findings indicate a potential role for mGlu5 receptors in mitigating the behavioral effects of 5-HT2AR agonists or modulating sensitivity to these agonists [151] (Figure 1). Recent studies by Burnat et al. [170] suggest that interactions between mGlu4 and 5-HT1AR receptors may represent another signaling pathway involved in the development and treatment of psychiatric disorders such as schizophrenia or depression. These findings highlight the importance of understanding the interactions between serotonin and metabotropic receptors in the context of neuropsychiatric diseases and their potential as therapeutic targets.

### 5.2. Pathophysiological Relevance for Cocaine Use Disorder and Potential Drug Development

It is of substantial interest that A2AR–D2R heterocomplexes play a major role in cocaine reward and addiction [150]. It was found that the A2AR agonist CGS21680 reduced cocaine reinforcement under a progressive ratio schedule, indicating that the A2AR activation diminishes the motivational effects of cocaine. Furthermore, upon disruption of the A2AR–D2R heterocomplex in the nucleus accumbens through a microinjection of A2AR transmembrane 5 peptide, an A2AR–D2R interface interfering peptide, the cocaine self-administration was enhanced by the ability of this peptide to set free the D2R protomer from allosteric A2AR-mediated inhibition [171,172]. In this way, evidence was obtained that A2AR–D2R heteroreceptor complexes, through their antagonistic allosteric receptor–receptor interactions, can be highly relevant targets for the treatment of cocaine self-administration. In agreement, the A2AR transmembrane 2 peptide, which is not part of the A2AR–D2R interface, failed to interfere with the A2AR–D2R heterocomplex as indicated by in situ PLA and lacked effects on the rat cocaine self-administration [171]. Thus, cocaine addiction development may depend on enhanced inhibition of D2R protomer signaling through increased allosteric A2AR protomer-mediated inhibition of the D2R protomer in the A2AR–D2R heteroreceptor complex. It is mainly located in the accumbens–pallidal GABA antireward neurons [150].

Furthermore, there exist indications that cocaine can recruit the intracellular Sigma1R to the plasma membrane [173], where it forms a heterocomplex with the D2R to which several Sigma1 R molecules can bind [174,175]. It was proposed that a trimeric complex of A2AR–D2R-Sigma1R can be formed in cocaine use disorder. It may be processed into a permanent irreversible molecular engram, which can strongly inhibit D2R protomer recognition and signaling in a long-lasting way, leading to cocaine addiction [150]. It takes place through a marked reduction of inhibitory D2R signaling in the GABA antireward neurons, leading to excess GABA antireward activity. In support of this hypothesis, combined treatment in cells expressing A2AR, D2R and Sigma 1R with cocaine and an A2AR agonist resulted in a dramatic reduction in the D2R agonist-induced activation of the Gi/o-mediated signaling as studied through inhibition of the activity of cAMP response element binding proteins (CREB) [150].

This concept is also supported by experiments on OSU-6162, a selective Sigma 1R ligand in low doses [176]. This Sigma1R ligand produced in the nucleus accumbens shell substantial increases in the density of the D2R–Sigma1R and A2AR–D2R heterocomplexes, supporting the existence of A2AR–D2R–Signa1R trimeric complexes in which the Sigma1R agonist can strongly enhance the antagonistic allosteric A2AR–D2R interaction. This mechanism may mediate the enhanced antagonistic A2AR–D2R interaction, causing marked inhibition of cocaine reward, leading to cocaine addiction.

### 5.3. Pathophysiological Relevance for Parkinson’s Disease and Drug Development

The A2AR–D2R and A1–D1R heterocomplexes modulating the key indirect and direct pathways of the basal ganglia are strongly implicated in Parkinson’s disease and its treatment [147]. These homo- and heteroreceptor complexes, including their antagonistic allosteric receptor–receptor interactions, play a key role in the basal ganglia. The A2AR–D2R complex modulates motor inhibition mediated by the indirect pathway, and the A1R–D1R complex modulates the motor initiation of the direct pathway. Ionotropic and metabotropic glutamate receptor protomers in heterocomplexes also participate in the regulation of these pathways. It has led to potentially improved treatment of Parkinson’s disease [147].

It is also suggested that A2AR and their heteroreceptor complexes, including the A2AR-alpha-synuclein complex ([177] and Borroto-Escuela et al. unpublished data), can have a relevant role in potentially increasing the propagation of alpha-synuclein monomers/dimers into oligomers and fibrils. Such events may also interfere with the function of various A2A heteroreceptor complexes including A2AR–D2R, A2AR–mGluR5, A2AR–mGluR1and A2AR-NMDAR contributing to deficits in learning and memory and enhanced neurodegeneration [134], involving especially the mGluR1 protomer [178,179]. It is proposed that the A2AR agonist, through activation of the A2AR protomer of the alpha-synuclein–A2AR homo and heterocomplex, may increase the alpha-synuclein propagation and produce disturbances in the A2A homo and heteroreceptor complexes interfering with learning and memory and increasing neurodegeneration through their dysfunction. In contrast, the A2AR antagonists like istradefylline can exert neuroprotective actions and improve cognition by, e.g., reducing the formation of alpha-synuclein–A2AR heterocomplexes and blocking the actions of A2AR agonists leading to reduced dysfunction [177].

Aggregates of misfolded alpha-synuclein are a hallmark of Parkinson’s disease. Misfolding is caused by mutations of the alpha-synuclein [180] and its degree likely enhances it. It should be investigated if the misfolding of the alpha-synuclein due to mutations will increase the formation of alpha-synuclein–A2AR heterocomplexes, which may enhance the disturbances they may induce in multiple A2A heteroreceptor complexes in the dorsal striatopallidal GABA neurons which upon their activation inhibit movements [181,182].

The alpha-synuclein complexes of various types may also be internalized into surrounding DA nerve terminals from, e.g., their release from dorsal striatal-pallidal GABA neurons via the vesicle mode of volume transmission. These events may start the degeneration of the striatal DA nerve terminals of the nigro-striatal DA neurons in Parkinson’s disease [147,177,183,184]. These neurons are highly vulnerable to Parkinson’s disease.

## 6. Conclusions and Future Aspects

One issue is to find the optimal nomenclature to describe the consequences of allosteric receptor–receptor interactions in the plasma membrane. The terms fine tuning or meta-modulation have been introduced to characterize receptor–receptor interactions in, e.g., heteroreceptor dimers or higher-order heteroreceptor complexes. However, in this article, the term protein modulation was introduced since the receptor complexes usually also contain not only receptor proteins but also other types of proteins like adaptor proteins. In future work, with increasing knowledge of the molecular composition of the heteroreceptor complexes and their associated allosteric receptor–receptor-protein interactions, it should be possible to find out if protein modulation would be a suitable term to describe the molecular changes in receptors and other proteins linked to changes in, e.g., cognition, signaling and/or trafficking.

Another issue will be to find the heteroreceptor complexes that are the most vulnerable in distinct CNS diseases like major depressive disorder, cocaine addiction and Parkinson’s disease, including the use of animal models of such diseases. It is proposed that it can lead to the development of novel treatment, provided distinct vulnerability can be identified in models of Parkinson’s disease.

## References

[1] Fuxe K., Agnati L.F., Benfenati F., Celani M., Zini I., Zoli M., Mutt V. (1983). Evidence for the Existence of Receptor Receptor Interactions in the Central Nervous-System—Studies on the Regulation of Monoamine Receptors by Neuropeptides. J. Neural Transm..

[2] Zoli M., Agnati L.F., Hedlund P.B., Li X.M., Ferre S., Fuxe K. (1993). Receptor-Receptor Interactions an Integrative Mechanism in Nerve-Cells. Mol. Neurobiol..

[3] Fuxe K., Agnati L.F., Borroto-Escuela D.O. (2014). The impact of receptor-receptor interactions in heteroreceptor complexes on brain plasticity. Expert Rev. Neurother..

[4] Borroto-Escuela D.O., Carlsson J., Ambrogini P., Narvaez M., Wydra K., Tarakanov A.O., Li X., Millon C., Ferraro L., Cuppini R. (2017). Understanding the Role of GPCR Heteroreceptor Complexes in Modulating the Brain Networks in Health and Disease. Front. Cell Neurosci..

[5] Fuxe K., Marcellino D., Borroto-Escuela D.O., Frankowska M., Ferraro L., Guidolin D., Ciruela F., Agnati L.F. (2010). The changing world of G protein-coupled receptors: From monomers to dimers and receptor mosaics with allosteric receptor-receptor interactions. J. Recept. Signal Transduct. Res..

[6] White J.H., Wise A., Main M.J., Green A., Fraser N.J., Disney G.H., Barnes A.A., Emson P., Foord S.M., Marshall F.H. (1998). Heterodimerization is required for the formation of a functional GABA(B) receptor. Nature.

[7] Marshall F.H., White J., Main M., Green A., Wise A. (1999). GABA(B) receptors function as heterodimers. Biochem. Soc. Trans..

[8] Kuhn C., Meyerhof W. (2013). Oligomerization of sweet and bitter taste receptors. Methods Cell Biol..

[9] Parker M.S., Park E.A., Sallee F.R., Parker S.L. (2011). Two intracellular helices of G-protein coupling receptors could generally support oligomerization and coupling with transducers. Amino Acids.

[10] Lee F.J., Xue S., Pei L., Vukusic B., Chery N., Wang Y., Wang Y.T., Niznik H.B., Yu X.M., Liu F. (2002). Dual regulation of NMDA receptor functions by direct protein-protein interactions with the dopamine D1 receptor. Cell.

[11] Borroto-Escuela D.O., Tarakanov A.O., Fuxe K. (2016). FGFR1-5-HT1A Heteroreceptor Complexes: Implications for Understanding and Treating Major Depression. Trends Neurosci..

[12] Kotliar I.B., Lorenzen E., Schwenk J.M., Hay D.L., Sakmar T.P. (2023). Elucidating the Interactome of G Protein-Coupled Receptors and Receptor Activity-Modifying Proteins. Pharmacol. Rev..

[13] Arapulisamy O., Mannangatti P., Jayanthi L.D. (2013). Regulated norepinephrine transporter interaction with the neurokinin-1 receptor establishes transporter subcellular localization. J. Biol. Chem..

[14] Liu F., Wan Q., Pristupa Z.B., Yu X.M., Wang Y.T., Niznik H.B. (2000). Direct protein-protein coupling enables cross-talk between dopamine D5 and gamma-aminobutyric acid A receptors. Nature.

[15] Perez de la Mora M., Ferre S., Fuxe K. (1997). GABA-dopamine receptor-receptor interactions in neostriatal membranes of the rat. Neurochem. Res..

[16] Flajolet M., Wang Z., Futter M., Shen W., Nuangchamnong N., Bendor J., Wallach I., Nairn A.C., Surmeier D.J., Greengard P. (2008). FGF acts as a co-transmitter through adenosine A(2A) receptor to regulate synaptic plasticity. Nat. Neurosci..

[17] Borroto-Escuela D.O., Romero-Fernandez W., Mudo G., Perez-Alea M., Ciruela F., Tarakanov A.O., Narvaez M., Di Liberto V., Agnati L.F., Belluardo N. (2012). Fibroblast Growth Factor Receptor 1- 5-Hydroxytryptamine 1A Heteroreceptor Complexes and Their Enhancement of Hippocampal Plasticity. Biol. Psychiatry.

[18] Fuxe K., Borroto-Escuela D.O. (2016). Heteroreceptor Complexes and their Allosteric Receptor-Receptor Interactions as a Novel Biological Principle for Integration of Communication in the CNS: Targets for Drug Development. Neuropsychopharmacology.

[19] Fuxe K., Borroto-Escuela D.O., Romero-Fernandez W., Palkovits M., Tarakanov A.O., Ciruela F., Agnati L.F. (2014). Moonlighting proteins and protein-protein interactions as neurotherapeutic targets in the G protein-coupled receptor field. Neuropsychopharmacology.

[20] Kolb P., Kenakin T., Alexander S.P.H., Bermudez M., Bohn L.M., Breinholt C.S., Bouvier M., Hill S.J., Kostenis E., Martemyanov K.A. (2022). Community guidelines for GPCR ligand bias: IUPHAR review 32. Br. J. Pharmacol..

[21] Kenakin T. (2011). Functional selectivity and biased receptor signaling. J. Pharmacol. Exp. Ther..

[22] Chini B. (2019). Expanding neuropeptide signalling by multiplying receptor functional states and sub-cellular locations. Cell Tissue Res..

[23] Borroto-Escuela D.O., Brito I., Romero-Fernandez W., Di Palma M., Oflijan J., Skieterska K., Duchou J., Van Craenenbroeck K., Suarez-Boomgaard D., Rivera A. (2014). The G protein-coupled receptor heterodimer network (GPCR-HetNet) and its hub components. Int. J. Mol. Sci..

[24] Perez de la Mora M., Borroto-Escuela D.O., Crespo-Ramirez M., Rejon-Orantes J.D.C., Palacios-Lagunas D.A., Martinez-Mata M.K., Sanchez-Luna D., Tesoro-Cruz E., Fuxe K. (2022). Dysfunctional Heteroreceptor Complexes as Novel Targets for the Treatment of Major Depressive and Anxiety Disorders. Cells.

[25] Borroto-Escuela D.O., DuPont C.M., Li X., Savelli D., Lattanzi D., Srivastava I., Narvaez M., Di Palma M., Barbieri E., Andrade-Talavera Y. (2017). Disturbances in the FGFR1-5-HT1A Heteroreceptor Complexes in the Raphe-Hippocampal 5-HT System Develop in a Genetic Rat Model of Depression. Front. Cell. Neurosci..

[26] Lefkowitz R.J. (2007). Seven transmembrane receptors: Something old, something new. Acta Physiol..

[27] Gurevich V.V., Gurevich E.V. (2008). GPCR monomers and oligomers: It takes all kinds. Trends Neurosci..

[28] Javitch J.A. (2004). The ants go marching two by two: Oligomeric structure of G-protein-coupled receptors. Mol. Pharmacol..

[29] Lee S.P., O’Dowd B.F., George S.R. (2003). Homo- and hetero-oligomerization of G protein-coupled receptors. Life Sci..

[30] Dale N.C., Johnstone E.K.M., Pfleger K.D.G. (2022). GPCR heteromers: An overview of their classification, function and physiological relevance. Front. Endocrinol..

[31] Han Y., Moreira I.S., Urizar E., Weinstein H., Javitch J.A. (2009). Allosteric communication between protomers of dopamine class A GPCR dimers modulates activation. Nat. Chem. Biol..

[32] Jordan B.A., Devi L.A. (1999). G-protein-coupled receptor heterodimerization modulates receptor function. Nature.

[33] Romero-Fernandez W., Borroto-Escuela D.O., Agnati L.F., Fuxe K. (2013). Evidence for the existence of dopamine D2-oxytocin receptor heteromers in the ventral and dorsal striatum with facilitatory receptor-receptor interactions. Mol. Psychiatry.

[34] Borroto-Escuela D.O., Li X., Tarakanov A.O., Savelli D., Narvaez M., Shumilov K., Andrade-Talavera Y., Jimenez-Beristain A., Pomierny B., Diaz-Cabiale Z. (2017). Existence of Brain 5-HT1A-5-HT2A Isoreceptor Complexes with Antagonistic Allosteric Receptor-Receptor Interactions Regulating 5-HT1A Receptor Recognition. ACS Omega.

[35] Terrillon S., Durroux T., Mouillac B., Breit A., Ayoub M.A., Taulan M., Jockers R., Barberis C., Bouvier M. (2003). Oxytocin and vasopressin V1a and V2 receptors form constitutive homo- and heterodimers during biosynthesis. Mol. Endocrinol..

[36] Kaupmann K., Malitschek B., Schuler V., Heid J., Froestl W., Beck P., Mosbacher J., Bischoff S., Kulik A., Shigemoto R. (1998). GABA(B)-receptor subtypes assemble into functional heteromeric complexes. Nature.

[37] Galvez T., Duthey B., Kniazeff J., Blahos J., Rovelli G., Bettler B., Prezeau L., Pin J.P. (2001). Allosteric interactions between GB1 and GB2 subunits are required for optimal GABA(B) receptor function. EMBO J..

[38] Milligan G., Ramsay D., Pascal G., Carrillo J.J. (2003). GPCR dimerisation. Life Sci..

[39] Rivero-Muller A., Chou Y.Y., Ji I., Lajic S., Hanyaloglu A.C., Jonas K., Rahman N., Ji T.H., Huhtaniemi I. (2010). Rescue of defective G protein-coupled receptor function in vivo by intermolecular cooperation. Proc. Natl. Acad. Sci. USA.

[40] Lee C., Ji I.J., Ji T.H. (2002). Use of defined-function mutants to access receptor-receptor interactions. Methods.

[41] Borroto-Escuela D.O., Flajolet M., Agnati L.F., Greengard P., Fuxe K. (2013). Bioluminescence resonance energy transfer methods to study G protein-coupled receptor-receptor tyrosine kinase heteroreceptor complexes. Methods Cell Biol..

[42] Schellekens H., De Francesco P.N., Kandil D., Theeuwes W.F., McCarthy T., van Oeffelen W.E., Perello M., Giblin L., Dinan T.G., Cryan J.F. (2015). Ghrelin’s Orexigenic Effect Is Modulated via a Serotonin 2C Receptor Interaction. ACS Chem. Neurosci..

[43] Yang J., Gong Z., Lu Y.B., Xu C.J., Wei T.F., Yang M.S., Zhan T.W., Yang Y.H., Lin L., Liu J. (2020). FLIM-FRET-Based Structural Characterization of a Class-A GPCR Dimer in the Cell Membrane. J. Mol. Biol..

[44] Herrick-Davis K., Grinde E., Cowan A., Mazurkiewicz J.E. (2013). Fluorescence correlation spectroscopy analysis of serotonin, adrenergic, muscarinic, and dopamine receptor dimerization: The oligomer number puzzle. Mol. Pharmacol..

[45] Herrick-Davis K., Grinde E., Lindsley T., Cowan A., Mazurkiewicz J.E. (2012). Oligomer size of the serotonin 5-hydroxytryptamine 2C (5-HT2C) receptor revealed by fluorescence correlation spectroscopy with photon counting histogram analysis: Evidence for homodimers without monomers or tetramers. J. Biol. Chem..

[46] Jonas K.C., Huhtaniemi I., Hanyaloglu A.C. (2016). Single-molecule resolution of G protein-coupled receptor (GPCR) complexes. Methods Cell Biol..

[47] Jonas K.C., Fanelli F., Huhtaniemi I.T., Hanyaloglu A.C. (2015). Single molecule analysis of functionally asymmetric G protein-coupled receptor (GPCR) oligomers reveals diverse spatial and structural assemblies. J. Biol. Chem..

[48] Qin G., Xu J., Liang Y., Fang X. (2023). Single-Molecule Imaging Reveals Differential AT1R Stoichiometry Change in Biased Signaling. Int. J. Mol. Sci..

[49] Pediani J.D., Ward R.J., Marsango S., Milligan G. (2018). Spatial Intensity Distribution Analysis: Studies of G Protein-Coupled Receptor Oligomerisation. Trends Pharmacol. Sci..

[50] Perreault M.L., Hasbi A., Shen M.Y.F., Fan T., Navarro G., Fletcher P.J., Franco R., Lanciego J.L., George S.R. (2016). Disruption of a dopamine receptor complex amplifies the actions of cocaine. Eur. Neuropsychopharm..

[51] Nai Q., Li S., Wang S.H., Liu J., Lee F.J., Frankland P.W., Liu F. (2009). Uncoupling the D1-N-methyl-D-aspartate (NMDA) receptor complex promotes NMDA-dependent long-term potentiation and working memory. Biol. Psychiatry.

[52] Cottet M., Faklaris O., Falco A., Trinquet E., Pin J.P., Mouillac B., Durroux T. (2013). Fluorescent ligands to investigate GPCR binding properties and oligomerization. Biochem. Soc. Trans..

[53] Cottet M., Albizu L., Comps-Agrar L., Trinquet E., Pin J.P., Mouillac B., Durroux T. (2011). Time resolved FRET strategy with fluorescent ligands to analyze receptor interactions in native tissues: Application to GPCR oligomerization. Methods Mol. Biol..

[54] Albizu L., Cottet M., Kralikova M., Stoev S., Seyer R., Brabet I., Roux T., Bazin H., Bourrier E., Lamarque L. (2010). Time-resolved FRET between GPCR ligands reveals oligomers in native tissues. Nat. Chem. Biol..

[55] Borroto-Escuela D.O., Craenenbroeck K.V., Romero-Fernandez W., Guidolin D., Woods A.S., Rivera A., Haegeman G., Agnati L.F., Tarakanov A.O., Fuxe K. (2010). Dopamine D2 and D4 receptor heteromerization and its allosteric receptor-receptor interactions. Biochem. Biophys. Res. Commun..

[56] Borroto-Escuela D.O., Romero-Fernandez W., Garriga P., Ciruela F., Narvaez M., Tarakanov A.O., Palkovits M., Agnati L.F., Fuxe K. (2013). G protein-coupled receptor heterodimerization in the brain. Methods Enzymol..

[57] Trifilieff P., Rives M.L., Urizar E., Piskorowski R.A., Vishwasrao H.D., Castrillon J., Schmauss C., Slattman M., Gullberg M., Javitch J.A. (2011). Detection of antigen interactions ex vivo by proximity ligation assay: Endogenous dopamine D2-adenosine A2A receptor complexes in the striatum. Biotechniques.

[58] Soderberg O., Gullberg M., Jarvius M., Ridderstrale K., Leuchowius K.J., Jarvius J., Wester K., Hydbring P., Bahram F., Larsson L.G. (2006). Direct observation of individual endogenous protein complexes in situ by proximity ligation. Nat. Methods.

[59] Raykova D., Kermpatsou D., Malmqvist T., Harrison P.J., Sander M.R., Stiller C., Heldin J., Leino M., Ricardo S., Klemm A. (2022). A method for Boolean analysis of protein interactions at a molecular level. Nat. Commun..

[60] Fuxe K., Borroto-Escuela D.O., Fuxe K., Borroto-Escuela D.O. (2018). Receptor-Receptor Interactions in the Central Nervous System.

[61] Romero-Fernandez W., Carvajal-Tapia C., Prusky A., Katdare K.A., Wang E., Shostak A., Ventura-Antunes L., Harmsen H.J., Lippmann E.S., Fuxe K. (2023). Detection, visualization and quantification of protein complexes in human Alzheimer’s disease brains using proximity ligation assay. Sci. Rep..

[62] Borroto-Escuela D.O., Lopez-Salas A., Wydra K., Bartolini M., Zhou Z., Frankowska M., Suder A., Benitez-Porres J., Romero-Fernandez W., Filip M. (2023). Combined treatment with Sigma1R and A2AR agonists fails to inhibit cocaine self-administration despite causing strong antagonistic accumbal A2AR-D2R complex interactions: The potential role of astrocytes. Front. Mol. Neurosci..

[63] Romero-Fernandez W., Taura J.J., Crans R.A.J., Lopez-Cano M., Fores-Pons R., Narvaez M., Carlsson J., Ciruela F., Fuxe K., Borroto-Escuela D.O. (2022). The mGlu5 Receptor Protomer-Mediated Dopamine D2 Receptor Trans-Inhibition Is Dependent on the Adenosine A2A Receptor Protomer: Implications for Parkinson’s Disease. Mol. Neurobiol..

[64] Chruscicka B., Cowan C.S.M., Wallace Fitzsimons S.E., Borroto-Escuela D.O., Druelle C.M., Stamou P., Bergmann C.A., Dinan T.G., Slattery D.A., Fuxe K. (2021). Molecular, biochemical and behavioural evidence for a novel oxytocin receptor and serotonin 2C receptor heterocomplex. Neuropharmacology.

[65] Chruscicka B., Wallace Fitzsimons S.E., Borroto-Escuela D.O., Druelle C., Stamou P., Nally K., Dinan T.G., Cryan J.F., Fuxe K., Schellekens H. (2019). Attenuation of Oxytocin and Serotonin 2A Receptor Signaling through Novel Heteroreceptor Formation. ACS Chem. Neurosci..

[66] Stocco E., Sfriso M.M., Borile G., Contran M., Barbon S., Romanato F., Macchi V., Guidolin D., De Caro R., Porzionato A. (2021). Experimental Evidence of A(2A)-D(2) Receptor-Receptor Interactions in the Rat and Human Carotid Body. Front. Physiol..

[67] Andrianarivelo A., Saint-Jour E., Pousinha P., Fernandez S.P., Petitbon A., De Smedt-Peyrusse V., Heck N., Ortiz V., Allichon M.C., Kappes V. (2021). Disrupting D1-NMDA or D2-NMDA receptor heteromerization prevents cocaine’s rewarding effects but preserves natural reward processing. Sci. Adv..

[68] Zhu Y., Dwork A.J., Trifilieff P., Javitch J.A. (2020). Detection of G Protein-Coupled Receptor Complexes in Postmortem Human Brain by Proximity Ligation Assay. Curr. Protoc. Neurosci..

[69] Biezonski D.K., Trifilieff P., Meszaros J., Javitch J.A., Kellendonk C. (2015). Evidence for limited D1 and D2 receptor coexpression and colocalization within the dorsal striatum of the neonatal mouse. J. Comp. Neurol..

[70] Martinez-Pinilla E., Rico A.J., Rivas-Santisteban R., Lillo J., Roda E., Navarro G., Lanciego J.L., Franco R. (2020). Expression of GPR55 and either cannabinoid CB(1) or CB(2) heteroreceptor complexes in the caudate, putamen, and accumbens nuclei of control, parkinsonian, and dyskinetic non-human primates. Brain Struct. Funct..

[71] Martinez-Pinilla E., Reyes-Resina I., Onatibia-Astibia A., Zamarbide M., Ricobaraza A., Navarro G., Moreno E., Dopeso-Reyes I.G., Sierra S., Rico A.J. (2014). CB1 and GPR55 receptors are co-expressed and form heteromers in rat and monkey striatum. Exp. Neurol..

[72] Walsh S.M., Mathiasen S., Christensen S.M., Fay J.F., King C., Provasi D., Borrero E., Rasmussen S.G.F., Fung J.J., Filizola M. (2018). Single Proteoliposome High-Content Analysis Reveals Differences in the Homo-Oligomerization of GPCRs. Biophys. J..

[73] Song W., Duncan A.L., Sansom M.S.P. (2021). Modulation of adenosine A2a receptor oligomerization by receptor activation and PIP(2) interactions. Structure.

[74] Kasai R.S., Ito S.V., Awane R.M., Fujiwara T.K., Kusumi A. (2018). The Class-A GPCR Dopamine D2 Receptor Forms Transient Dimers Stabilized by Agonists: Detection by Single-Molecule Tracking. Cell Biochem. Biophys..

[75] Kasai R.S., Kusumi A. (2014). Single-molecule imaging revealed dynamic GPCR dimerization. Curr. Opin. Cell Biol..

[76] Kasai R.S., Suzuki K.G., Prossnitz E.R., Koyama-Honda I., Nakada C., Fujiwara T.K., Kusumi A. (2011). Full characterization of GPCR monomer-dimer dynamic equilibrium by single molecule imaging. J. Cell Biol..

[77] Chakraborty H., Jafurulla M., Clayton A.H.A., Chattopadhyay A. (2018). Exploring oligomeric state of the serotonin(1A) receptor utilizing photobleaching image correlation spectroscopy: Implications for receptor function. Faraday Discuss..

[78] Teichmann A., Gibert A., Lampe A., Grzesik P., Rutz C., Furkert J., Schmoranzer J., Krause G., Wiesner B., Schulein R. (2014). The specific monomer/dimer equilibrium of the corticotropin-releasing factor receptor type 1 is established in the endoplasmic reticulum. J. Biol. Chem..

[79] Marsango S., Jenkins L., Pediani J.D., Bradley S.J., Ward R.J., Hesse S., Biener G., Stoneman M.R., Tobin A.B., Raicu V. (2022). The M(1) muscarinic receptor is present in situ as a ligand-regulated mixture of monomers and oligomeric complexes. Proc. Natl. Acad. Sci. USA.

[80] Pediani J.D., Ward R.J., Godin A.G., Marsango S., Milligan G. (2016). Dynamic Regulation of Quaternary Organization of the M1 Muscarinic Receptor by Subtype-selective Antagonist Drugs. J. Biol. Chem..

[81] Park P.S., Wells J.W. (2003). Monomers and oligomers of the M2 muscarinic cholinergic receptor purified from Sf9 cells. Biochemistry.

[82] Petersen J., Wright S.C., Rodriguez D., Matricon P., Lahav N., Vromen A., Friedler A., Stromqvist J., Wennmalm S., Carlsson J. (2017). Agonist-induced dimer dissociation as a macromolecular step in G protein-coupled receptor signaling. Nat. Commun..

[83] Nguyen K.D.Q., Vigers M., Sefah E., Seppala S., Hoover J.P., Schonenbach N.S., Mertz B., O’Malley M.A., Han S. (2021). Homo-oligomerization of the human adenosine A(2A) receptor is driven by the intrinsically disordered C-terminus. eLife.

[84] Kharche S.A., Sengupta D. (2020). Dynamic protein interfaces and conformational landscapes of membrane protein complexes. Curr. Opin. Struct. Biol..

[85] Bockaert J., Marin P., Dumuis A., Fagni L. (2003). The ‘magic tail’ of G protein-coupled receptors: An anchorage for functional protein networks. FEBS Lett..

[86] Petit-Pedrol M., Groc L. (2021). Regulation of membrane NMDA receptors by dynamics and protein interactions. J. Cell Biol..

[87] Calebiro D., Koszegi Z. (2019). The subcellular dynamics of GPCR signaling. Mol. Cell. Endocrinol..

[88] Borroto-Escuela D.O., Fuxe K. (2019). Oligomeric Receptor Complexes and Their Allosteric Receptor-Receptor Interactions in the Plasma Membrane Represent a New Biological Principle for Integration of Signals in the CNS. Front. Mol. Neurosci..

[89] Lefkowitz R.J. (2004). Historical review: A brief history and personal retrospective of seven-transmembrane receptors. Trends Pharmacol. Sci..

[90] Franco R., Martinez-Pinilla E., Lanciego J.L., Navarro G. (2016). Basic Pharmacological and Structural Evidence for Class A G-Protein-Coupled Receptor Heteromerization. Front. Pharmacol..

[91] Hasbi A., O’Dowd B.F., George S.R. (2011). Dopamine D1-D2 receptor heteromer signaling pathway in the brain: Emerging physiological relevance. Mol. Brain.

[92] Maroteaux L., Bechade C., Roumier A. (2019). Dimers of serotonin receptors: Impact on ligand affinity and signaling. Biochimie.

[93] Ciruela F., Burgueno J., Casado V., Canals M., Marcellino D., Goldberg S.R., Bader M., Fuxe K., Agnati L.F., Lluis C. (2004). Combining mass spectrometry and pull-down techniques for the study of receptor heteromerization. Direct epitope-epitope electrostatic interactions between adenosine A2A and dopamine D2 receptors. Anal. Chem..

[94] Borroto-Escuela D.O., Marcellino D., Narvaez M., Flajolet M., Heintz N., Agnati L., Ciruela F., Fuxe K. (2010). A serine point mutation in the adenosine A2AR C-terminal tail reduces receptor heteromerization and allosteric modulation of the dopamine D2R. Biochem. Biophys. Res. Commun..

[95] Borroto-Escuela D.O., Rodriguez D., Romero-Fernandez W., Kapla J., Jaiteh M., Ranganathan A., Lazarova T., Fuxe K., Carlsson J. (2018). Mapping the Interface of a GPCR Dimer: A Structural Model of the A2A Adenosine and D2 Dopamine Receptor Heteromer. Front. Pharmacol..

[96] Levoye A., Dam J., Ayoub M.A., Guillaume J.L., Jockers R. (2006). Do orphan G-protein-coupled receptors have ligand-independent functions? New insights from receptor heterodimers. EMBO Rep..

[97] Bueschbell B., Manga P., Penner E., Schiedel A.C. (2021). Evidence for Protein-Protein Interaction between Dopamine Receptors and the G Protein-Coupled Receptor 143. Int. J. Mol. Sci..

[98] Levoye A., Dam J., Ayoub M.A., Guillaume J.L., Couturier C., Delagrange P., Jockers R. (2006). The orphan GPR50 receptor specifically inhibits MT1 melatonin receptor function through heterodimerization. EMBO J..

[99] Balenga N.A., Martinez-Pinilla E., Kargl J., Schroder R., Peinhaupt M., Platzer W., Balint Z., Zamarbide M., Dopeso-Reyes I.G., Ricobaraza A. (2014). Heteromerization of GPR55 and cannabinoid CB2 receptors modulates signalling. Br. J. Pharmacol..

[100] Reyes-Resina I., Navarro G., Aguinaga D., Canela E.I., Schoeder C.T., Zaluski M., Kiec-Kononowicz K., Saura C.A., Muller C.E., Franco R. (2018). Molecular and functional interaction between GPR18 and cannabinoid CB(2) G-protein-coupled receptors. Relevance in neurodegenerative diseases. Biochem. Pharmacol..

[101] Szymanska K., Kalafut J., Przybyszewska A., Paziewska B., Adamczuk G., Kielbus M., Rivero-Muller A. (2018). FSHR Trans-Activation and Oligomerization. Front. Endocrinol..

[102] Quitterer U., AbdAlla S. (2019). Discovery of Pathologic GPCR Aggregation. Front. Med..

[103] AbdAlla S., Lother H., Quitterer U. (2000). AT1-receptor heterodimers show enhanced G-protein activation and altered receptor sequestration. Nature.

[104] Gill K.S., Mehta K., Heredia J.D., Krishnamurthy V.V., Zhang K., Procko E. (2023). Multiple mechanisms of self-association of chemokine receptors CXCR4 and CCR5 demonstrated by deep mutagenesis. J. Biol. Chem..

[105] Contento R.L., Molon B., Boularan C., Pozzan T., Manes S., Marullo S., Viola A. (2008). CXCR4-CCR5: A couple modulating T cell functions. Proc. Natl. Acad. Sci. USA.

[106] Andrianarivelo A., Saint-Jour E., Walle R., Trifilieff P., Vanhoutte P. (2019). Modulation and functions of dopamine receptor heteromers in drugs of abuse-induced adaptations. Neuropharmacology.

[107] Liu X.Y., Chu X.P., Mao L.M., Wang M., Lan H.X., Li M.H., Zhang G.C., Parelkar N.K., Fibuch E.E., Haines M. (2006). Modulation of D2R-NR2B interactions in response to cocaine. Neuron.

[108] Tanganelli S., Antonelli T., Tomasini M.C., Beggiato S., Fuxe K., Ferraro L. (2012). Relevance of dopamine D(2)/neurotensin NTS1 and NMDA/neurotensin NTS1 receptor interaction in psychiatric and neurodegenerative disorders. Curr. Med. Chem..

[109] Marino M.J., Rouse S.T., Levey A.I., Potter L.T., Conn P.J. (1998). Activation of the genetically defined m1 muscarinic receptor potentiates N-methyl-D-aspartate (NMDA) receptor currents in hippocampal pyramidal cells. Proc. Natl. Acad. Sci. USA.

[110] Altier C., Zamponi G.W. (2011). Analysis of GPCR/ion channel interactions. Methods Mol. Biol..

[111] Hermosilla T., Moreno C., Itfinca M., Altier C., Armisen R., Stutzin A., Zamponi G.W., Varela D. (2011). L-type calcium channel beta subunit modulates angiotensin II responses in cardiomyocytes. Channels.

[112] Ambrogini P., Lattanzi D., Pagliarini M., Di Palma M., Sartini S., Cuppini R., Fuxe K., Borroto-Escuela D.O. (2023). 5HT1AR-FGFR1 Heteroreceptor Complexes Differently Modulate GIRK Currents in the Dorsal Hippocampus and the Dorsal Raphe Serotonin Nucleus of Control Rats and of a Genetic Rat Model of Depression. Int. J. Mol. Sci..

[113] Doupnik C.A. (2008). GPCR-Kir channel signaling complexes: Defining rules of engagement. J. Recept. Signal Transduct. Res..

[114] Sahlholm K., Nilsson J., Marcellino D., Fuxe K., Arhem P. (2007). The human histamine H3 receptor couples to GIRK channels in Xenopus oocytes. Eur. J. Pharmacol..

[115] Chen R., Ferris M.J., Wang S. (2020). Dopamine D2 autoreceptor interactome: Targeting the receptor complex as a strategy for treatment of substance use disorder. Pharmacol. Ther..

[116] Quarta D., Ciruela F., Patkar K., Borycz J., Solinas M., Lluis C., Franco R., Wise R.A., Goldberg S.R., Hope B.T. (2007). Heteromeric nicotinic acetylcholine-dopamine autoreceptor complexes modulate striatal dopamine release. Neuropsychopharmacology.

[117] Bontempi L., Savoia P., Bono F., Fiorentini C., Missale C. (2017). Dopamine D3 and acetylcholine nicotinic receptor heteromerization in midbrain dopamine neurons: Relevance for neuroplasticity. Eur. Neuropsychopharmacol. J. Eur. Coll. Neuropsychopharmacol..

[118] Di Palma M., Sartini S., Lattanzi D., Cuppini R., Pita-Rodriguez M., Diaz-Carmenate Y., Narvaez M., Fuxe K., Borroto-Escuela D.O., Ambrogini P. (2020). Evidence for the existence of A2AR-TrkB heteroreceptor complexes in the dorsal hippocampus of the rat brain: Potential implications of A2AR and TrkB interplay upon ageing. Mech. Ageing Dev..

[119] Narvaez M., Andrade-Talavera Y., Valladolid-Acebes I., Fredriksson M., Siegele P., Hernandez-Sosa A., Fisahn A., Fuxe K., Borroto-Escuela D.O. (2020). Existence of FGFR1-5-HT1AR heteroreceptor complexes in hippocampal astrocytes. Putative link to 5-HT and FGF2 modulation of hippocampal gamma oscillations. Neuropharmacology.

[120] Di Liberto V., Borroto-Escuela D.O., Frinchi M., Verdi V., Fuxe K., Belluardo N., Mudo G. (2017). Existence of muscarinic acetylcholine receptor (mAChR) and fibroblast growth factor receptor (FGFR) heteroreceptor complexes and their enhancement of neurite outgrowth in neural hippocampal cultures. Biochim. Biophys. Acta Gen. Subj..

[121] Fuxe K., Ferre S., Zoli M., Agnati L.F. (1998). Integrated events in central dopamine transmission as analyzed at multiple levels. Evidence for intramembrane adenosine A(2A) dopamine D-2 and adenosine A(1) dopamine D-1 receptor interactions in the basal ganglia. Brain Res. Rev..

[122] Torvinen M., Marcellino D., Canals M., Agnati L.F., Lluis C., Franco R., Fuxe K. (2005). Adenosine A2A receptor and dopamine D3 receptor interactions: Evidence of functional A2A/D3 heteromeric complexes. Mol. Pharmacol..

[123] Fuxe K., Marcellino D., Borroto-Escuela D.O., Guescini M., Fernandez-Duenas V., Tanganelli S., Rivera A., Ciruela F., Agnati L.F. (2010). Adenosine-dopamine interactions in the pathophysiology and treatment of CNS disorders. CNS Neurosci. Ther..

[124] Franco R. (2009). Neurotransmitter receptor heteromers in neurodegenerative diseases and neural plasticity. J. Neural Transm..

[125] George S.R., Kern A., Smith R.G., Franco R. (2014). Dopamine receptor heteromeric complexes and their emerging functions. Prog. Brain Res..

[126] Artigas F. (2013). Serotonin receptors involved in antidepressant effects. Pharmacol. Ther..

[127] Borroto-Escuela D.O., Romero-Fernandez W., Tarakanov A.O., Marcellino D., Ciruela F., Agnati L.F., Fuxe K. (2010). Dopamine D2 and 5-hydroxytryptamine 5-HT((2)A) receptors assemble into functionally interacting heteromers. Biochem. Biophys. Res. Commun..

[128] Kolasa M., Solich J., Faron-Gorecka A., Zurawek D., Pabian P., Lukasiewicz S., Kusmider M., Szafran-Pilch K., Szlachta M., Dziedzicka-Wasylewska M. (2018). Paroxetine and Low-dose Risperidone Induce Serotonin 5-HT(1A) and Dopamine D2 Receptor Heteromerization in the Mouse Prefrontal Cortex. Neuroscience.

[129] Borroto-Escuela D.O., Romero-Fernandez W., Narvaez M., Oflijan J., Agnati L.F., Fuxe K. (2014). Hallucinogenic 5-HT2AR agonists LSD and DOI enhance dopamine D2R protomer recognition and signaling of D2-5-HT2A heteroreceptor complexes. Biochem. Biophys. Res. Commun..

[130] Lukasiewicz S., Blasiak E., Szafran-Pilch K., Dziedzicka-Wasylewska M. (2016). Dopamine D2 and serotonin 5-HT1A receptor interaction in the context of the effects of antipsychotics—In vitro studies. J. Neurochem..

[131] Borroto-Escuela D.O., Ambrogini P., Narvaez M., Di Liberto V., Beggiato S., Ferraro L., Fores-Pons R., Alvarez-Contino J.E., Lopez-Salas A., Mudo G. (2021). Serotonin Heteroreceptor Complexes and Their Integration of Signals in Neurons and Astroglia-Relevance for Mental Diseases. Cells.

[132] Fuxe K., Agnati L.F., Cintra A., Andersson K., Eneroth P., Harfstrand A., Zoli M., Goldstein M. (1988). Studies on central D1 receptors role in volume transmission, neuroendrocrine regulation and release of noradrenaline. Adv. Exp. Med. Biol..

[133] Bjelke B., Stromberg I., O’Connor W.T., Andbjer B., Agnati L.F., Fuxe K. (1994). Evidence for volume transmission in the dopamine denervated neostriatum of the rat after a unilateral nigral 6-OHDA microinjection. Studies with systemic D-amphetamine treatment. Brain Res..

[134] Borroto-Escuela D.O., Agnati L.F., Bechter K., Jansson A., Tarakanov A.O., Fuxe K. (2015). The role of transmitter diffusion and flow versus extracellular vesicles in volume transmission in the brain neural-glial networks. Philos. Trans. R. Soc. Lond. B Biol. Sci..

[135] Fuxe K., Borroto-Escuela D.O. (2016). Volume transmission and receptor-receptor interactions in heteroreceptor complexes: Understanding the role of new concepts for brain communication. Neural Regen. Res..

[136] Agnati L.F., Cortelli P., Biagini G., Bjelke B., Fuxe K. (1994). Different classes of volume transmission signals exist in the central nervous system and are affected by metabolic signals, temperature gradients and pressure waves. Neuroreport.

[137] Katz P.S., Edwards D.H., Katz P.S. (1999). Metamodulation: The control and modulation of neuromodulationGet accessArrow. Beyond Neurotransmission: Neuromodulation and Its Importance for Information Processing.

[138] Philpot B.D., Bear M.F., Abraham W.C., Katz P.S. (1999). Metaplasticity: The plasticity of synaptic plasticity. Beyond Neurotransmission: Neuromodulation and Its Importance for Information Processing.

[139] Fuxe K., Agnati L.F. (1985). Receptor-receptor interactions in the central nervous system. A new integrative mechanism in synapses. Med. Res. Rev..

[140] Ribeiro J.A., Sebastiao A.M. (2010). Modulation and metamodulation of synapses by adenosine. Acta Physiol..

[141] Sebastiao A.M., Ribeiro J.A. (2009). Tuning and fine-tuning of synapses with adenosine. Curr. Neuropharmacol..

[142] Sebastiao A.M., Ribeiro J.A. (2015). Neuromodulation and metamodulation by adenosine: Impact and subtleties upon synaptic plasticity regulation. Brain Res..

[143] Fields R.D., Burnstock G. (2006). Purinergic signalling in neuron-glia interactions. Nat. Reviews. Neurosci..

[144] Ciruela F., Casado V., Rodrigues R.J., Lujan R., Burgueno J., Canals M., Borycz J., Rebola N., Goldberg S.R., Mallol J. (2006). Presynaptic control of striatal glutamatergic neurotransmission by adenosine A1-A2A receptor heteromers. J. Neurosci. Off. J. Soc. Neurosci..

[145] Cristovao-Ferreira S., Navarro G., Brugarolas M., Perez-Capote K., Vaz S.H., Fattorini G., Conti F., Lluis C., Ribeiro J.A., McCormick P.J. (2013). A1R-A2AR heteromers coupled to Gs and G i/0 proteins modulate GABA transport into astrocytes. Purinergic Signal..

[146] Borroto-Escuela D.O., Wydra K., Fores-Pons R., Vasudevan L., Romero-Fernandez W., Frankowska M., Ferraro L., Beggiato S., Crespo-Ramirez M., Rivera A. (2021). The Balance of MU-Opioid, Dopamine D2 and Adenosine A2A Heteroreceptor Complexes in the Ventral Striatal-Pallidal GABA Antireward Neurons May Have a Significant Role in Morphine and Cocaine Use Disorders. Front. Pharmacol..

[147] Borroto-Escuela D.O., Fuxe K. (2019). Adenosine heteroreceptor complexes in the basal ganglia are implicated in Parkinson’s disease and its treatment. J. Neural Transm..

[148] Iglesias A., Cimadevila M., Cadavid M.I., Loza M.I., Brea J. (2017). Serotonin-2A homodimers are needed for signalling via both phospholipase A2 and phospholipase C in transfected CHO cells. Eur. J. Pharmacol..

[149] Romero-Fernandez W., Wydra K., Borroto-Escuela D.O., Jastrzebska J., Zhou Z., Frankowska M., Filip M., Fuxe K. (2022). Increased density and antagonistic allosteric interactions in A2AR-D2R heterocomplexes in extinction from cocaine use, lost in cue induced reinstatement of cocaine seeking. Pharmacol. Biochem. Behav..

[150] Borroto-Escuela D.O., Wydra K., Filip M., Fuxe K. (2018). A2AR-D2R Heteroreceptor Complexes in Cocaine Reward and Addiction. Trends Pharmacol. Sci..

[151] Halberstadt A.L., Lehmann-Masten V.D., Geyer M.A., Powell S.B. (2011). Interactive effects of mGlu5 and 5-HT2A receptors on locomotor activity in mice. Psychopharmacology.

[152] Fuxe K., Agnati L.F., Marcoli M., Borroto-Escuela D.O. (2015). Volume Transmission in Central Dopamine and Noradrenaline Neurons and Its Astroglial Targets. Neurochem. Res..

[153] Taddeucci A., Olivero G., Roggeri A., Milanese C., Giorgio F.P.D., Grilli M., Marchi M., Garrone B., Pittaluga A. (2022). Presynaptic 5-HT(2A)-mGlu2/3 Receptor-Receptor Crosstalk in the Prefrontal Cortex: Metamodulation of Glutamate Exocytosis. Cells.

[154] Pittaluga A., Marchi M. (2022). Synaptosomes and Metamodulation of Receptors. Methods Mol. Biol..

[155] Fuxe K., Borroto-Escuela D.O., Ciruela F., Guidolin D., Agnati L.F. (2014). Receptor-receptor interactions in heteroreceptor complexes: A new principle in biology. Focus on their role in learning and memory. Neurosci. Discov..

[156] Pittaluga A., Roggeri A., Vallarino G., Olivero G. (2021). Somatostatin, a Presynaptic Modulator of Glutamatergic Signal in the Central Nervous System. Int. J. Mol. Sci..

[157] Agnati L.F., Fuxe K., Zoli M., Rondanini C., Ogren S.O. (1982). New vistas on synaptic plasticity: The receptor mosaic hypothesis of the engram. Med. Biol..

[158] Borroto-Escuela D.O., Wydra K., Pintsuk J., Narvaez M., Corrales F., Zaniewska M., Agnati L.F., Franco R., Tanganelli S., Ferraro L. (2016). Understanding the Functional Plasticity in Neural Networks of the Basal Ganglia in Cocaine Use Disorder: A Role for Allosteric Receptor-Receptor Interactions in A2A-D2 Heteroreceptor Complexes. Neural Plast..

[159] Uhlhaas P.J., Singer W. (2006). Neural synchrony in brain disorders: Relevance for cognitive dysfunctions and pathophysiology. Neuron.

[160] Borroto-Escuela D.O., Ambrogini P., Chruscicka B., Lindskog M., Crespo-Ramirez M., Hernandez-Mondragon J.C., Perez de la Mora M., Schellekens H., Fuxe K. (2021). The Role of Central Serotonin Neurons and 5-HT Heteroreceptor Complexes in the Pathophysiology of Depression: A Historical Perspective and Future Prospects. Int. J. Mol. Sci..

[161] Insel T.R., Young L.J. (2001). The neurobiology of attachment. Nat. Rev. Neurosci..

[162] Young L.J., Lim M.M., Gingrich B., Insel T.R. (2001). Cellular mechanisms of social attachment. Horm. Behav..

[163] Schellekens H., Dinan T.G., Cryan J.F. (2013). Taking two to tango: A role for ghrelin receptor heterodimerization in stress and reward. Front. Neurosci..

[164] De la Mora M.P., Perez-Carrera D., Crespo-Ramirez M., Tarakanov A., Fuxe K., Borroto-Escuela D.O. (2016). Signaling in dopamine D2 receptor-oxytocin receptor heterocomplexes and its relevance for the anxiolytic effects of dopamine and oxytocin interactions in the amygdala of the rat. Biochim. Biophys. Acta.

[165] Albizu L., Moreno J.L., Gonzalez-Maeso J., Sealfon S.C. (2010). Heteromerization of G protein-coupled receptors: Relevance to neurological disorders and neurotherapeutics. CNS Neurol. Disord. Drug Targets.

[166] Gonzalez-Maeso J., Ang R.L., Yuen T., Chan P., Weisstaub N.V., Lopez-Gimenez J.F., Zhou M., Okawa Y., Callado L.F., Milligan G. (2008). Identification of a serotonin/glutamate receptor complex implicated in psychosis. Nature.

[167] Baki L., Fribourg M., Younkin J., Eltit J.M., Moreno J.L., Park G., Vysotskaya Z., Narahari A., Sealfon S.C., Gonzalez-Maeso J. (2016). Cross-signaling in metabotropic glutamate 2 and serotonin 2A receptor heteromers in mammalian cells. Pflug. Arch. Eur. J. Physiol..

[168] Moreno J.L., Miranda-Azpiazu P., Garcia-Bea A., Younkin J., Cui M., Kozlenkov A., Ben-Ezra A., Voloudakis G., Fakira A.K., Baki L. (2016). Allosteric signaling through an mGlu2 and 5-HT2A heteromeric receptor complex and its potential contribution to schizophrenia. Sci. Signal..

[169] Moreno J.L., Muguruza C., Umali A., Mortillo S., Holloway T., Pilar-Cuellar F., Mocci G., Seto J., Callado L.F., Neve R.L. (2012). Identification of three residues essential for 5-hydroxytryptamine 2A-metabotropic glutamate 2 (5-HT2A.mGlu2) receptor heteromerization and its psychoactive behavioral function. J. Biol. Chem..

[170] Burnat G., Branski P., Solich J., Kolasa M., Chruscicka B., Dziedzicka-Wasylewska M., Pilc A. (2020). The functional cooperation of 5-HT(1A) and mGlu4R in HEK-293 cell line. Pharmacol. Rep..

[171] Borroto-Escuela D.O., Wydra K., Romero-Fernandez W., Zhou Z., Frankowska M., Filip M., Fuxe K. (2019). A2AR Transmembrane 2 Peptide Administration Disrupts the A2AR-A2AR Homoreceptor but Not the A2AR-D2R Heteroreceptor Complex: Lack of Actions on Rodent Cocaine Self-Administration. Int. J. Mol. Sci..

[172] Borroto-Escuela D.O., Wydra K., Li X., Rodriguez D., Carlsson J., Jastrzebska J., Filip M., Fuxe K. (2018). Disruption of A2AR-D2R Heteroreceptor Complexes After A2AR Transmembrane 5 Peptide Administration Enhances Cocaine Self-Administration in Rats. Mol. Neurobiol..

[173] Kourrich S., Su T.P., Fujimoto M., Bonci A. (2012). The sigma-1 receptor: Roles in neuronal plasticity and disease. Trends Neurosci..

[174] Beggiato S., Borelli A.C., Borroto-Escuela D., Corbucci I., Tomasini M.C., Marti M., Antonelli T., Tanganelli S., Fuxe K., Ferraro L. (2017). Cocaine modulates allosteric D2-sigma1 receptor-receptor interactions on dopamine and glutamate nerve terminals from rat striatum. Cell. Signal..

[175] Borroto-Escuela D.O., Narvaez M., Wydra K., Pintsuk J., Pinton L., Jimenez-Beristain A., Di Palma M., Jastrzebska J., Filip M., Fuxe K. (2017). Cocaine self-administration specifically increases A2AR-D2R and D2R-sigma1R heteroreceptor complexes in the rat nucleus accumbens shell. Relevance for cocaine use disorder. Pharmacol. Biochem. Behav..

[176] Borroto-Escuela D.O., Romero-Fernandez W., Wydra K., Zhou Z., Suder A., Filip M., Fuxe K. (2020). OSU-6162, a Sigma1R Ligand in Low Doses, Can Further Increase the Effects of Cocaine Self-Administration on Accumbal D2R Heteroreceptor Complexes. Neurotox. Res..

[177] Borroto-Escuela D.O., Fuxe K. (2022). The integrative role of G protein-coupled receptor heterocomplexes in Parkinson’s disease. Neural Regen. Res..

[178] Aguirre J.A., Andbjer B., Gonzalez-Baron S., Hansson A., Stromberg I., Agnati L.F., Fuxe K. (2001). Group I mGluR antagonist AIDA protects nigral DA cells from MPTP-induced injury. Neuroreport.

[179] Yamasaki T., Fujinaga M., Kawamura K., Furutsuka K., Nengaki N., Shimoda Y., Shiomi S., Takei M., Hashimoto H., Yui J. (2016). Dynamic Changes in Striatal mGluR1 But Not mGluR5 during Pathological Progression of Parkinson’s Disease in Human Alpha-Synuclein A53T Transgenic Rats: A Multi-PET Imaging Study. J. Neurosci. Off. J. Soc. Neurosci..

[180] Ferreira D.G., Batalha V.L., Vicente Miranda H., Coelho J.E., Gomes R., Goncalves F.Q., Real J.I., Rino J., Albino-Teixeira A., Cunha R.A. (2017). Adenosine A2A Receptors Modulate alpha-Synuclein Aggregation and Toxicity. Cereb. Cortex.

[181] Sulzer D., Surmeier D.J. (2013). Neuronal vulnerability, pathogenesis, and Parkinson’s disease. Mov. Disord. Off. J. Mov. Disord. Soc..

[182] Surmeier D.J., Obeso J.A., Halliday G.M. (2017). Selective neuronal vulnerability in Parkinson disease. Nat. Rev. Neurosci..

[183] Perez de la Mora M., Hernandez-Mondragon C., Crespo-Ramirez M., Rejon-Orantes J., Borroto-Escuela D.O., Fuxe K. (2020). Conventional and Novel Pharmacological Approaches to Treat Dopamine-Related Disorders: Focus on Parkinson’s Disease and Schizophrenia. Neuroscience.

[184] Borroto-Escuela D.O., Perez De La Mora M., Manger P., Narvaez M., Beggiato S., Crespo-Ramirez M., Navarro G., Wydra K., Diaz-Cabiale Z., Rivera A. (2018). Brain Dopamine Transmission in Health and Parkinson’s Disease: Modulation of Synaptic Transmission and Plasticity Through Volume Transmission and Dopamine Heteroreceptors. Front. Synaptic Neurosci..

